# The crosstalk between lymphatic endothelial cells and immune cells in Physiological and Pathological conditions

**DOI:** 10.1007/s00018-026-06228-8

**Published:** 2026-06-10

**Authors:** Weijie Chen, Yongheng Wang, Da Zhang, Yifan Zhang, Xixi Qin, Xiaolei Wang

**Affiliations:** Department of Gastroenterology, Shanghai Tenth People’s Hospital, School of Medicine, Tongji University, Shanghai, 200072 China

**Keywords:** Lymphatic vessel, Leukocyte, Immunomodulation, Cell interaction, Inflammation, Tumor microenvironment

## Abstract

The lymphatic system serves two primary functions: the absorption of interstitial fluid and the coordination of immune cell interactions and transport. Central to these roles is the dynamic interplay between lymphatic endothelial cells (LECs) and immune cells, which acts as a critical hub for maintaining physiological homeostasis and regulating pathological processes. Under physiological conditions, LECs orchestrate immune responses by establishing chemokine gradients, modulating the metabolic microenvironment, and expressing adhesion molecules. They collaborate with dendritic cells (DCs) to initiate immunity, finely regulate the differentiation and homing of T cell subsets, support B cell-mediated humoral immunity, and work with macrophages to maintain tissue homeostasis. In pathological states, LECs assume a dual role. In inflammatory diseases, such as infections and autoimmune disorders, they recruit immune cells through the secretion of pro-inflammatory cytokines to clear pathogens; conversely, their aberrant antigen presentation may break immune tolerance and promote the activation of autoreactive T and B cells. During cancer progression, tumor-associated LECs adopt context-dependent programs, either constructing immunosuppressive networks that facilitate immune evasion, lymphatic metastasis and resistance to immunotherapy, or supporting antigen transport and presentation that promote antitumor immunity. This review systematically elucidates the molecular mechanisms and translational implications of the interactions between LECs and immune cells. It provides a theoretical foundation for precision therapies targeting the lymphatic-immune network. The insights garnered not only enhance our understanding of the mechanisms underlying disease pathogenesis but also unveil novel therapeutic avenues for targeting inflammatory diseases and cancer, holding significant value for both basic research and clinical translation.

## Introduction

The lymphatic system, a vital component of the vascular system, is responsible for the critical functions of interstitial fluid reabsorption, lipid transport, and immune cell trafficking [1]. Its reticular network of lymphatic vessels and lymph nodes (LNs) forms a sophisticated infrastructure that not only clears pathogens but also enables systemic immune surveillance through the circulation of immune cells [2]. Lymphatic endothelial cells, the monolayer of flattened epithelial cells lining the lymphatic lumen, exhibit remarkable structural and functional heterogeneity. Their junctional architecture dynamically transitions between “button-like junctions” in capillary lymphatic vessels (allowing macromolecule uptake) and “zipper-like junctions” in collecting lymphatic vessels (maintaining luminal integrity) [3, 4]. Furthermore, LECs utilize the CDC42/RhoA signaling pathway to remodel their cytoskeleton, adopting an “oak leaf-like” morphology that confers exceptional resilience against mechanical stress from interstitial fluid pressure fluctuations [5].

More importantly, LECs transcend their drainage role to emerge as a pivotal hub for immune regulation. They secrete chemokines to guide the homing of dendritic cells and T cells, thereby initiating adaptive immunity [6, 7]. Within pathological microenvironments, LECs display functional heterogeneity. In inflammatory states, they play a dual role: recruiting immune cells to clear pathogens, while their aberrant antigen presentation can potentially break peripheral tolerance [8, 9]. In cancer, tumor-associated LECs (TA-LECs) construct an immunosuppressive network that facilitates tumor immune evasion and metastasis [10–12]. Recent single-cell and multi-omics approaches have further revealed that LECs are not a homogeneous population but comprise multiple transcriptionally and spatially distinct subsets with organ- and niche-specific immune functions [13–15]. Deciphering the spatiotemporal dynamics of the crosstalk between LECs and immune cells provides not only novel perspectives for elucidating the pathogenesis of inflammation, autoimmune diseases, and cancer but also paves the way for developing precision interventional strategies. Targeting chemokine receptors, immune checkpoints, or metabolic pathways within the lymphatic network holds significant promise for reshaping the inflammatory microenvironment and overcoming resistance to cancer immunotherapy.

However, most previous studies have focused either on lymphatic vessels themselves or on the trafficking of single immune cell types, without systematically comparing how LECs coordinate different leukocyte subsets across conditions. This review therefore integrates current knowledge on LEC interactions with DCs, T cells, B cells, neutrophils and macrophages along capillary and collecting lymphatics, highlighting shared principles and context-dependent differences in homeostasis, infection, autoimmunity and cancer. By bridging vascular biology and immunology, we aim to provide a unifying framework for LEC–immune cell crosstalk and to identify conceptual and translational gaps that may guide future research.

## Dynamic crosstalk between lymphatic endothelial cells and dendritic cells

The interaction between LECs and DCs serves as a pivotal bridge connecting local tissue immunity with systemic adaptive responses. This crosstalk exquisitely regulates the initiation and resolution of immune responses through mechanisms involving chemokine gradients, adhesion molecule-mediated migration, and antigen transfer/tolerance induction. The function of this dynamic interaction undergoes significant reprogramming within inflammatory and tumor microenvironments, profoundly influencing disease progression.

### LEC-DC Interactions under physiological homeostasis

**Fig. 1:**
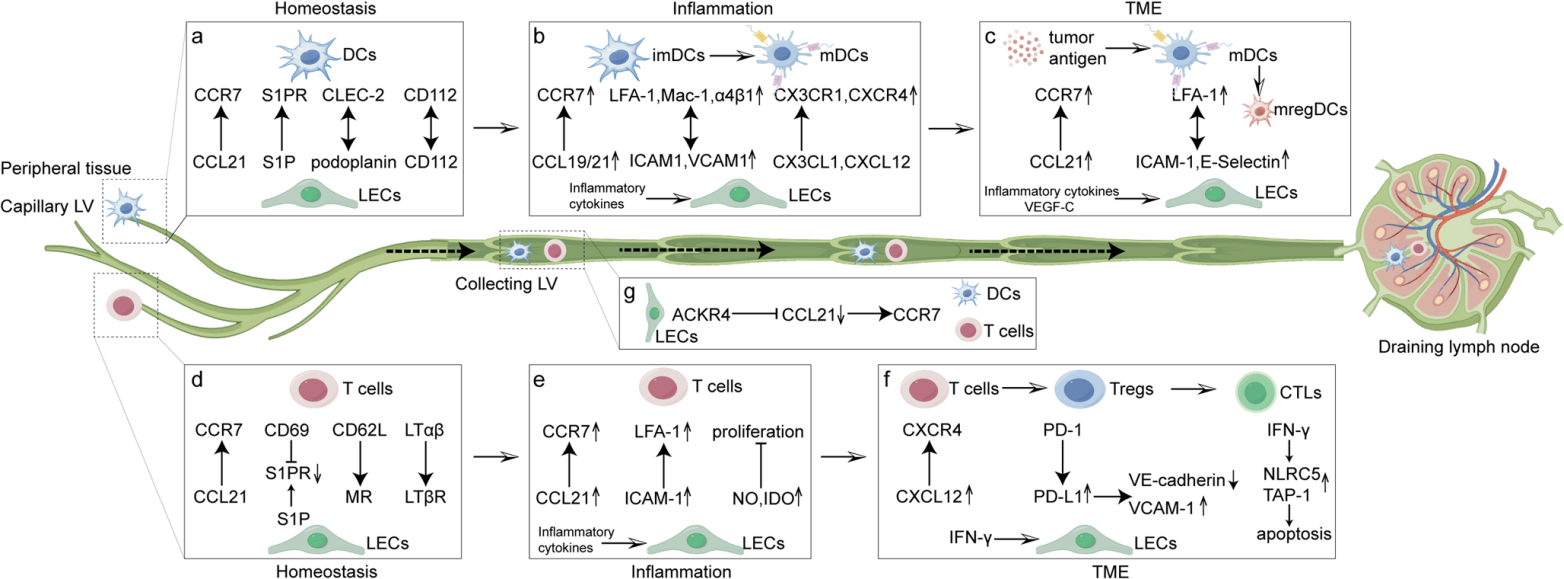
Mechanisms of lymphatic endothelial cells interactions with dendritic cells and T cells in homeostasis, inflammation, and cancer. (**a**) Under homeostatic conditions, LECs express CCL21 to guide CCR7^+^ DCs towards capillary LVs. DCs docking and transmigration are facilitated by interactions including podoplanin/CLEC-2, S1P/S1PR and CD112/CD112 [18, 21–23]. (**b**) During inflammation, mature DCs upregulate CCR7, integrins (LFA-1, Mac-1), and chemokine receptors (CX3CR1, CXCR4). Inflamed LECs enhance the expression of CCL19/CCL21, adhesion molecules (ICAM-1, VCAM-1), and chemokines (CX3CL1, CXCL12), collectively promoting DC recruitment and trafickingto draining lymph nodes [24, 25, 26, 27]. (**c**) In the tumor microenvironment, LECs upregulate CCL21 and adhesion molecules (ICAM-1, E-selectin), facilitating the migration of conventional DCs and mregDCs that capture tumor antigens [28–30]. (**d**) Under homeostatic conditions, T cell migration into lymphatic capillaries is primarily guided by CCL21-CCR7. This process is also finely regulated by molecular signals such as S1P/S1PR, CD62L/MR, and LTαβ/LTβR [31–34]. (**e**) During inflammation, upregulated CCL21 and ICAM-1 on LECs enhance CCR7^+^ T cell recruitment and LFA-1-dependent intraluminal crawling. Simultaneously, LECs can suppress T cell proliferation by releasing mediators like NO and IDO [35–39]. (**f**) In the TME, LECs secrete CXCL12 to engage CXCR4 on T cells, potentially excluding them from the tumor core. Under IFN-γ stimulation, LECs express up-regulated PD-L1 that interacts with PD-1 on T cells, inhibiting CD8 + T cell activation and promoting Tregs transendothelial migration by modulating VE-cadherin and VCAM-1. Conversely, upon sensing high levels of IFN-γ from CTLs, LECs can upregulate antigen-cross-presentation machinery (NLRC5, TAP1), making them targets for CTL-mediated killing [40–42]. (**g**) Lymphatic endothelial cells induce expression of ACKR4 in response to fluid shear stress, actively clearing CCR7 ligands (CCL19/CCL21) from the luminal surface to facilitate the transition of DCs and T cells from active migration to passive streaming transport [43]. Abbreviations:*LV* lymphatic vessel, *DCs* Dendritic cells, *CCR7* Chemokine (C-C motif) receptor 7, *CCL21* chemokine (C-C motif) ligand 21, *S1P* Sphingosine-1-Phosphate, *S1PR* Sphingosine-1-phosphate receptor, *CLEC-2* C-type Lectin-like Receptor 2, *LECs* Lymphatic endothelial cells, *LFA-1* Lymphocyte Function-associated Antigen-1, *Mac-1* Macrophage antigen-1, *ICAM-1* Intercellular adhesion molecule 1‌, *VCAM-1* Vascular cell adhesion molecule-1, *CX3CL1* chemokine (C-X3-C motif) ligand 1, *CXCL12* Chemokine (C-X-C motif) ligand 12, *CX3CR1* Chemokine (C-X3-C motif) receptor 1‌, *CXCR4* chemokine (C-X-C motif) receptor 4, *TME* tumor microenvironment‌, *mDCs* mature dendritic cells, *mregDCs* mature regulatory dendritic cells, *MR* Macrophage Mannose Receptor, *LTαβ* Lymphotoxin α and β‌, *LTβR* lymphotoxin β receptor, *NO* nitric oxide, *IDO* indoleamine 2,3-dioxygenase, *Tregs* Regulatory T cells‌, *CTLs* Cytotoxic T Lymphocytes, *PD-1* programmed death-1, *PD-L1* programmed death ligand 1, *VE-cadherin* vascular endothelial-cadherin *IFN*-γ Interferon γ, *NLRC5* NLR family CARD domain containing 5‌, *TAP-1* transporter associated with antigen processing 1. Figure was created with figdraw.com and Adobe Illustrator

Under healthy conditions, LECs orchestrate DCs migration and the initiation of adaptive immunity through multiple mechanisms, thereby maintaining immune homeostasis between tissues and lymphoid organs. These concepts build on early mechanistic work in skin-lymph cannulation models, which provided direct evidence that antigen-bearing DCs exit peripheral tissues via afferent lymphatic vessels to reach draining lymph nodes [16]. Together with the seminal conceptual framework proposed by Randolph and colleagues, who integrated dendritic-cell trafficking with lymphatic vascular biology and CCR7–CCL21 chemokine guidance [17], these studies established afferent lymphatic migration as a central paradigm for DC egress that underpins the mechanisms discussed below. Firstly, LECs express the chemokine CCL21, establishing a chemical gradient within tissues that guides the directional migration of mature DCs expressing the CCR7 receptor toward lymphatic vessels [18] (Fig. 1a). Notably, CCL19, another ligand for CCR7, appears to be dispensable for the lymphatic migration of DCs under physiological homeostasis [19]. Interestingly, this process is regulated by circadian rhythms; CCL21 expression in skin LECs peaks during the murine rest phase (daytime) and at night in humans, synchronously enhancing the efficiency of DCs entry into lymphatic vessels and optimizing the rhythm of immune surveillance [20].

Upon reaching capillary lymphatic vessels (characterized by endothelial cells with “button-like” junctions forming openable flap valves [4]) DCs dock onto the basolateral surface of the lymphatic endothelium. Here, surface hyaluronan (HA) on DCs interacts with lymphatic vessel endothelial hyaluronan receptor 1 (LYVE-1) on LECs, forming dynamic “transmigratory cups” that facilitate their entry from the interstitium into the lymphatic lumen [44]. Concurrently, adhesion molecules on LECs (e.g., ICAM-1, podoplanin) bind to integrins Mac-1 (αMβ2) and LFA-1 (αLβ2) expressed by DCs. These interactions not only strengthen DCs adhesion to the endothelium [45] but also trigger actin reorganization and active contraction within DCs, enabling them to squeeze through basement membrane pores and push aside endothelial flaps to complete transendothelial migration [46, 47]. Once inside the capillary lymphatic vessels, DCs are not merely passive passengers in the flow. They undergo semi-directed crawling (approximately 30% of their path toward the draining lymph node) via ROCK kinase-mediated contractile movement [48, 49]. This intraluminal crawling remains dependent on the CCL21 concentration gradient expressed by LECs and is stabilized by ICAM-1-mediated adhesion. It is important to note, however, that in the absence of inflammatory stimuli, lymphatic vessels in vivo express very low levels of ICAM-1 and VCAM-1 compared to their in vitro cultured counterparts [50]. When DCs enter collecting lymphatic vessels, their migration becomes predominantly passive, driven by the flow of lymph [51, 52].

Although CCL21/CCR7 signaling is paramount for DCs migration to LNs, emerging research indicates contributions from other chemotactic pathways at various steps of this process. For instance, LECs are considered a primary source of the high sphingosine-1-phosphate (S1P) levels found in lymph. Mature DCs have been shown to upregulate S1P receptors and exhibit chemotaxis toward S1P [21]. Beyond chemokines, the small transmembrane glycoprotein podoplanin, expressed by LECs, interacts with the C-type lectin receptor CLEC-2 on DCs, thereby promoting DCs migration to draining lymph nodes (dLNs) [22]. Most recently, it was discovered that both human dermal DCs and LECs highly express CD112. Upon contact with the lymphatic endothelium, CD112 on the DCs surface directly engages with CD112 on LECs, forming homodimeric adhesive junctions that enhance the initial anchoring of DCs to the vessel wall [23] (Fig. 1a). Simultaneously, bidirectional signaling is continuously active within this interaction. Contact from DCs can provide feedback to LECs, contributing to the maintenance of lymphatic vessel structure. In vivo imaging and genetic models have confirmed that CCR7^+^ IRF4^+^ dendritic cells, through sustained contact with LECs, directly regulate the basal permeability of collecting lymphatic vessels, preventing lymph leakage and subsequent fibrosis [53].

Importantly, CCR7 is not restricted to DCs but is also highly expressed on T cells [31]. Thus, the CCL21 gradients established by LECs preconfigure the migratory landscape not only for antigen-bearing DCs but also for CCR7⁺ T cells, foreshadowing the tightly coupled DC–T cell circuit discussed in later sections.

### LEC-DC Interactions under inflammatory conditions

The inflammatory microenvironment reprograms the phenotypes of both LECs and DCs, altering their interaction patterns and conferring a “double-edged sword” characteristic in infections, tissue injury, and autoimmune diseases.

In infectious diseases, the interplay between LECs and DCs functions to enhance antigen clearance and immune initiation. Stimulated by inflammatory and pathogen-derived signals, immature DCs differentiate into mature conventional DCs (cDCs), with significantly upregulated expression of MHC class II molecules, co-stimulatory molecules, and the chemokine receptor CCR7 [54, 55]. CCR7 guides the migration of cDCs via afferent lymphatic vessels to LNs by interacting with LEC-derived CCL19 and CCL21 [24, 25] (Fig. 1b). In TNF-α-, LPS- or IL-1β-driven cutaneous inflammation, CD137 signaling on LECs increases CCL21 production and thereby enhances CCR7-dependent DC entry into lymphatics [24], whereas in viral infection models CCL21 can be downregulated and leukocyte trafficking to lymph nodes instead relies on Ch25h-mediated oxysterol cues [25]. These findings underscore that modulation of CCL21 during inflammation is highly context-dependent. In the subcapsular sinus (SCS) region of the lymph node, DCs continue their migration along a CCL21 concentration gradient formed by the expression of the atypical chemokine scavenger receptor ACKR4 on the outer wall (roof) of the SCS [56]. After crossing the inner wall (floor) of the SCS, DCs finally enter the T cell zone in a CCR7-dependent manner to interact with naïve T cells [57].

Beyond CCL19 and CCL21, numerous other chemokines and cytokines participate in regulating DCs migration under inflammation. LECs upregulate the expression of adhesion molecules ICAM-1, VCAM-1, and E-selectin. They also produce chemokine C-X3-C motif ligand 1 (CX3CL1) and CXCL12, which bind to the chemokine receptors CX3CR1 and CXCR4 on the DCs surface, further promoting DCs migration towards lymphatic vessels [26, 58]. It is important to note, however, that in the absence of pathogens but under inflammatory stimulation such as TNF-α, direct contact between DCs and the inflamed lymphatic endothelium leads to a downregulation of the co-stimulatory molecule CD86 on DCs. This specific mechanism is mediated by the interaction between macrophage-1 antigen (Mac-1) on DCs and ICAM-1 on LECs, suggesting a role for the lymphatic endothelium in preventing excessive immune responses during inflammation [27] (Fig. 1b).

Although LEC-DC interactions serve a defensive role in infections, acute infections or prolonged stimulation from chronic inflammation can also impair the function of both LECs and DCs. On the endothelial side, sustained inflammatory signaling has been reported to disrupt LEC junctional integrity and impair lymphatic pumping, thereby compromising lymphatic drainage and leukocyte trafficking [59]. On the dendritic-cell side, for example, during SARS-CoV-2 infection, plasmacytoid dendritic cells (pDCs), conventional type 1 dendritic cells (cDC1s), and type 2 dendritic cells (cDC2s) all showed reduced expression levels of CD80, CD86, CCR7, and HLA-DR after stimulation with Toll-like receptor (TLR) ligands, indicating that the maturation and migration capacity of DC subsets may be compromised following acute SARS-CoV-2 infection [60].

Chemokines and their receptors involved in various LEC-DC interactions are also closely associated with the pathogenesis and progression of autoimmune diseases, including multiple sclerosis (MS), systemic lupus erythematosus (SLE), rheumatoid arthritis (RA), Hashimoto’s thyroiditis (HT), and inflammatory bowel diseases (IBD) [61–64]. Elevated expression levels of DC-related chemokines and their receptors have been detected in the blood, synovial fluid, and synovial tissue of RA patients, with some of these molecules serving as potential biomarkers for disease activity/severity [65]. Preclinical trials targeting the chemokine system for RA treatment have shown significant promise in early stages [62]. CCR7-mediated DC migration plays a crucial role in the development of collagen-induced arthritis (CIA, a mouse model of RA). The immunomodulator FTY720 significantly reduces the incidence and severity of CIA by downregulating CCR7 expression in DCs [66]. Treatment with an anti-human CCR7 monoclonal antibody in humanized CCR7 mice not only prevented the induction of CIA but also suppressed disease progression [67]. Furthermore, recent studies have revealed that the natural compound saikosaponin D can block the migration of dendritic cells induced by the RA-specific autoantigen type II collagen by targeting and inhibiting Optineurin protein expression, thereby enhancing the ubiquitination modification of the CCR7 receptor and promoting its proteasomal degradation [68]. These studies confirm CCR7 as a potential therapeutic target for RA and other autoimmune diseases.

Similarly, in the disease process of Hashimoto’s thyroiditis, intrathyroidal LECs and myofibroblasts co-express CCL19/CCL21, forming tertiary lymphoid structures that recruit CCR7^+^ DCs to the lesion site. Within the inflammatory microenvironment, DCs present high levels of thyroglobulin (Tg), activating autoreactive T cells and driving the destruction of thyroid follicles [63]. It is noteworthy that in the research on targeting the CCL19/CCL21-CCR7 axis to intervene in DC migration for treating autoimmune diseases, the current focus heavily lies on regulating the CCR7 expression on DCs. In contrast, studies targeting the expression of chemokines on LECs remain relatively scarce. This insight inspires further in-depth investigation in this latter direction.

During the pathological progression of inflammatory bowel disease, remodeling of the lymphatic system has been observed, ranging from capillary lymphatics to mesenteric collecting lymphatics and ultimately to mesenteric lymph nodes. Compared with control groups, both ulcerative colitis (UC) and Crohn’s disease (CD) patients exhibit increased lymphangiogenesis, lymphatic vessel dilation, and elevated expression of lymphangiogenic markers within the lymphatic vessels [69–71]. In the *TNF*^*ΔARE*^ mouse model of Crohn’s disease, an increased number of tertiary lymphoid organs (TLOs) associated with mesenteric collecting lymphatic vessels was found to mechanically obstruct DC transport, leading to local immune activation [72]. Our previous single-cell transcriptomic study of the creeping fat in Crohn’s disease patients revealed significantly upregulated expression of the CD74 gene in LECs derived from the creeping fat of CD patients [73]. Intriguingly, in DCs, CD74 negatively regulates their migration speed by modulating the localization and activity of myosin II, thereby spatiotemporally coordinating antigen processing with cell motility [74]. Therefore, whether the high expression of CD74 in LECs impacts the migration of DCs within lymphatic vessels warrants further investigation.

Taken together, these studies support a unifying model in which inflammatory cues reprogram both LECs and DCs, transiently enhancing CCR7-dependent trafficking and antigen delivery to LNs while simultaneously installing negative feedback loops that restrain excessive activation. However, chronic or dysregulated activation of the same pathways appears to facilitate the recruitment of autoreactive DCs, thereby driving autoimmune pathology. This duality highlights a central conceptual tension: mechanisms that are protective in acute infection can become pathogenic when inflammation is spatially or temporally uncontrolled. Future work comparing DC–LEC crosstalk across organs and across disease stages will be crucial to understand when targeting the CCL19/CCL21–CCR7 axis is likely to restore tolerance versus impair host defense.

### LEC-DC Interactions in the tumor microenvironment

Although inflammatory infiltration is a key feature of tumorigenesis and development, the tumor microenvironment (TME) represents a unique, pathological inflammatory state. This environment is fundamentally and markedly distinct from classic inflammatory environments aimed at pathogen clearance and tissue repair, in terms of cellular composition, functional purpose, molecular signaling networks, and ultimate outcome [75–77]. Therefore, this review discusses the interactions between LECs and immune cells within the TME separately.

Metastasis of tumors to regional draining lymph nodes is considered an early event that predicts poor prognosis in various solid tumors [78, 79]. Consequently, high-risk patients undergo sentinel lymph node biopsy during tumor resection to determine their staging and guide clinical treatment strategies. Tumor-associated lymphatic vessels (TA-LVs) provide an essential pathway for lymphatic metastasis, and as the tumor progresses, lymphatic vessel density often increases due to lymphangiogenesis [80]. Increasing evidence suggests that tumor-associated lymphatic vessel formation plays a significant dual role in tumor immunity [81]. As key components of the TME, lymphatic vessels can promote antitumor immune responses by enhancing immune cell infiltration and antigen presentation, as shown in melanoma models with peritumoral lymphangiogenesis and in glioblastoma where VEGF-C-driven meningeal lymphatics facilitate T-cell priming and response to anti-PD-1 therapy [82, 83]; yet, particularly in melanoma, they may also suppress effective immune surveillance by facilitating immune evasion and lymph node metastasis [84]. Together, these findings underscore the complexity of tumor-associated lymphangiogenesis in cancer progression and in shaping responses to immunotherapy.

TA-LVs play a pivotal role in activating adaptive immunity. Within the TME, lymphatic vessels enhance the migratory capacity of DCs. VEGF-C and inflammatory cytokines can upregulate CCL21 expression. In the tumor microenvironment, inflammatory cytokines such as TNF-α and prostaglandin E2 upregulate the expression of CCR7 on DCs, which respond to the upregulated CCL21 by LECs to migrate from tumor or inflammatory tissues to lymphatic vessels and draining lymph nodes [28, 85]. Soluble tumor-derived antigens, immune complexes and extracellular vesicles can drain with interstitial fluid and enter lymphatic vessels directly, adding a second, passive route of antigen delivery [86, 87]. These antigens are subsequently captured and processed by DCs and macrophages within the dLNs, which then activate CD8^+^ T cells and CD4^+^ T cells via major histocompatibility complex class I (MHC-I) or class II (MHC-II) molecules, respectively [88]. Multiple adhesion molecules also regulate DCs entry into afferent lymphatic vessels. In the inflammatory tumor microenvironment, LECs upregulate adhesion molecules such as intercellular adhesion molecule-1 (ICAM-1) and E-selectin, which serve as ligands for integrins like lymphocyte function-associated antigen-1 (LFA-1) on the surface of DCs [29] (Fig. 1c).

Despite their capacity to promote immune activation, TA-LVs can also exert multiple negative effects during tumor progression, including facilitating metastasis, inducing immune tolerance, and assisting in immune evasion [89].As mentioned previously, the adhesive interaction between migratory dendritic cells and LECs exposed to inflammatory cytokines can downregulate DC activation levels and their stimulatory capacity through MAC-1/ICAM-1 engagement [27]. Furthermore, LECs in VEGF-C–overexpressing inflamed skin can release anti-inflammatory prostacyclin to inhibit dendritic cell activation [90]. However, the inhibitory effects of tumor-associated lymphatic endothelial cells on dendritic cells remain to be further investigated.

The rapid development of single-cell sequencing technologies has unveiled numerous novel immune cell subtypes. Recent extensive characterization of tumor myeloid cells using single-cell technology revealed that intratumoral LAMP3^+^ DCs express gene-encoded factors associated with both immune regulation (PD-L1, PD-L2, and CD200) and migration/maturation (CD40, CCR7, and IL-12), leading to their designation as mregDCs [30] (Fig. 1c). Further studies using quantitative multiplex imaging demonstrated that mregDCs, via high expression of the migration receptor CCR7, respond to CCL21 secreted by lymphatic endothelial cells. This interaction enables their specific localization to perivascular areas of lymphatic vessels within the tumor stroma, forming an immunosuppressive microenvironment composed of regulatory T cells (Tregs), mregDCs, and lymphatic vessels. This niche hinders tumor immune surveillance and promotes tumor progression [91]. Notably, since their definition, the current understanding of mregDCs primarily originates from tumor immunology studies [30, 92, 93]. The role of LEC-mregDC interactions in a broader disease context remains to be elucidated and warrants in-depth exploration.

## Multifaceted interactions between lymphatic endothelial cells and T lymphocytes

The interactions between lymphatic endothelial cells and T lymphocytes play a central role in immune homeostasis, inflammatory responses, and tumor progression. These interactions are orchestrated through chemokine gradients, adhesion molecules, and metabolic signaling, demonstrating dynamic alterations across various pathological states.

### LEC-T Cell interactions under physiological homeostasis: Precise regulation of lymphocyte trafficking and immune homeostasis

Cannulation studies conducted in animals and healthy humans have demonstrated that under homeostatic conditions, T lymphocytes constitute the majority (80–90%) of the cellular composition within afferent lymph [94, 95]. It is noteworthy that most T cells trafficking through afferent lymphatic vessels are antigen-experienced CD4^+^ effector memory T cells (TEMs). These cells are believed to recirculate from peripheral tissues back into the bloodstream, thereby fulfilling their immune surveillance functions. As naïve T cells primarily migrate between the blood and secondary lymphoid organs (SLOs) and typically lack the homing molecules required for extravasation into peripheral tissues, they are present only in small numbers within afferent lymph. CD8^+^ T cells are also present in afferent lymph, though their numbers are significantly lower compared to CD4^+^ TEMs [96–98]. Studies utilizing photoconvertible transgenic mouse models have further revealed that regulatory T cells (Tregs) represent a significant CD4^+^ T cell subset within afferent lymph flow, comprising 20–25% under steady-state conditions and up to 50% during inflammatory states [96, 99, 100].

Similar to DCs, CCR7 plays a crucial role in the migratory process of T cells. CCR7 guides not only the egress of T cells from peripheral tissues but also their entry into draining lymphatic vessels [31]. By contrast, the S1P/S1PR1 axis has more complex and apparently context-dependent effects on T-cell trafficking. In peripheral tissues, activation of S1PR1 can inhibit CCR7-dependent entry of T cells into afferent lymphatic vessels, thereby favoring their retention in the tissue [101]. In other settings, however, S1PR1 signaling is best known for promoting lymphocyte egress, and transcriptional downregulation of S1pr1 is required for the establishment and maintenance of tissue-resident memory CD8⁺ T cells [102]. Consistently, the activation marker CD69 physically interacts with S1PR1, induces its internalization and degradation, and in this way interferes with S1P-mediated egress signals to stabilize T-cell retention and TRM formation in peripheral tissues [32] (Fig. 1d). Thus, whether the S1P/S1PR1 axis promotes tissue retention or egress remains a matter of debate and likely depends on the specific T-cell subset involved (e.g. tissue-resident versus recirculating memory T cells), and this issue requires further investigation.

Furthermore, other molecules expressed on lymphatic vessels have been reported to participate in lymphocyte migration regulation: Compared to vascular endothelial cells, lymphatic endothelial cells specifically express the macrophage mannose receptor (MR). By directly binding to ligands on the lymphocyte surface (such as L-selectin or glycosylated proteins), MR mediates the transmigration of lymphocytes across the lymphatic barrier and can simultaneously regulate the migration of CD4⁺ T cells, CD8⁺ T cells, and B cells to dLNs [33]. Lymphatic endothelial cells can also sense the LTαβ signal expressed by Tregs via the lymphotoxin beta receptor (LTβR). This activates the non-canonical NF-κB pathway (NIK-dependent), induces VCAM-1 expression and cytoskeletal remodeling, thereby actively mediating the transendothelial migration of Tregs and their specific drainage to the LNs [34] (Fig. 1d). Additionally, common lymphatic endothelial and vascular endothelial receptor-1 (CLEVER-1) is also important for T cell entry into afferent lymphatic vessels. Blocking CLEVER-1 significantly reduces the migration of both CD4^+^ and CD8^+^ T cells from the skin to the dLNs [103]. Interestingly, subsequent studies discovered that CLEVER-1 can also mediate the migration of dendritic cells to dLNs [104], which again highlights the inseparable collaborative relationship between DCs and T cells.

Beyond guiding T cell migration, LECs are directly involved in regulating T cell activation. Under homeostatic conditions, LECs constitutively express MHC class I and II molecules, enabling them to present both self-antigens and exogenous peptides captured from the lymph. However, due to low expression of HLA-DM, which impedes CLIP removal [105], and the inhibition of cathepsin activity by cystatins (Cystatin C/B) [106], their antigen processing efficiency is suboptimal. Furthermore, LECs lack the co-stimulatory molecules CD80 and CD86 [107]. Consequently, while LECs can present antigenic signals to T cells, they are unable to deliver effective activation signals. Conversely, LECs express PD-L1 and MHC class II (the latter serving as a ligand for LAG-3). Through the PD-1/PD-L1 and LAG-3/MHC-II pathways, they induce deletional tolerance in CD8⁺ T cells [107] and promote CD4⁺ T cell tolerance via antigen presentation, collectively maintaining peripheral immune homeostasis [108]. It must be emphasized that this tolerance phenotype characterizes lymphoepithelial cells under steady-state conditions. In disease states, any contribution of LECs to loss of tolerance in disease is likely to occur indirectly—for example by modulating DC maturation, cytokine milieus or checkpoint pathways—rather than via classical co-stimulatory activation [109–111].

### LEC-T Cell interactions under inflammatory conditions

As previously mentioned, in several inflammatory models, LECs significantly upregulate CCL21 expression to enhance the migration of cDCs [35]. However, besides DCs, almost all migratory T cells are also CCR7-positive [36]. Consequently, the upregulation of the CCL21-CCR7 axis also enhances the efficient egress of CCR7^+^ T cells from peripheral tissues affected by acute inflammation (Fig. 1e). After the adoptive transfer of CCR7-deficient splenocytes into skin inflamed by Complete Freund’s Adjuvant (CFA), the migration rate of CD4^+^ T cells was reduced by approximately 80% [112]. Targeting this pathway to promote the migration of inflammatory T cells has been proven to alleviate the progression of acute inflammation. For instance, in the acute inflammatory response following subarachnoid hemorrhage (SAH), enhancing the drainage function of meningeal lymphatic vessels for Th17 cells via the CCR7-CCL21 pathway reduced brain injury and improved behavioral outcomes in a SAH mouse model [113]. Similarly, inflammatory cytokines such as TNF-α and IFN-γ can directly stimulate LECs to upregulate the adhesion molecule ICAM-1, thereby promoting the active crawling of T cells in an ICAM-1/LFA-1-dependent manner [37] (Fig. 1e).

Interestingly, although the high luminal concentration of CCL21 in lymphatic vessels promotes T cell migration from peripheral tissues into capillary lymphatic vessels, it also leads to excessive T cell adhesion under inflammatory conditions, which impedes their entry into collecting lymphatic vessels. A study using dermal inflammation induced by topical application of TPA revealed that, to counter this, during inflammation, endothelial cells of collecting lymphatic vessels upregulate the expression of the atypical chemokine receptor ACKR4 in response to fluid shear stress. This receptor actively scavenges CCR7 ligands (CCL19/CCL21) from the luminal surface, thereby reducing integrin-mediated adhesion strength of T cells. This process facilitates the transition of T cells from active migration to passive flow transport, ultimately ensuring their efficient drainage to the LNs [43] (Fig. 1g). Therefore, combined with the findings on the migration mechanism of DCs during inflammation (Sect. 2.2), the current findings highlight that CCR7-dependent migration forms an integrated circuit comprising both DCs and T cells. CCR7 guides antigen-loaded DCs from peripheral tissues to draining LNs and simultaneously positions naïve and memory T cells within the T-cell zone. Consequently, many in vivo phenotypes observed in CCR7- or CCL19/CCL21-deficient mice arise from concerted defects in both DC and T-cell trafficking, and cannot readily be attributed to a single cell type unless cell-type–specific interventions are used.

LECs can also function as a “reservoir” for foreign antigens during infection. Following viral challenge or vaccination, under conditions that induce lymphatic endothelial cell proliferation, antigens can be captured and persist within LECs for several weeks. This persistence enhances the production of IFN-γ and interleukin-2 (IL-2), positively modulating the level of protective immunity provided by circulating memory CD8^+^ T cells and improving anti-infection protective efficacy [110].However, this antigen “reservoir” function of LECs can have detrimental effects in certain infectious diseases. For instance, in extrapulmonary tuberculosis, the most common sites of infection are located within the lymphatic system. LECs have been revealed to serve as a key replicative niche for Mycobacterium tuberculosis, facilitating its growth through the region of difference 1 (RD1)-dependent cytosolic escape and exploitation of autophagosomes. This suggests that LECs may act as a potential reservoir for persistent tuberculosis infection and relapse [114]. In the context of HIV infection, LECs directly promote HIV infection and the formation of latent reservoirs in resting CD4⁺ T cells by secreting IL-6. Furthermore, they significantly enhance the infection efficiency of activated CD4⁺ T cells without relying on MHC II-TCR or CD2-CD58 interactions [115]. These findings provide new perspectives for further understanding the establishment of bacterial and viral latent reservoirs and for developing therapeutic strategies targeting the lymphoid tissue microenvironment.

In autoimmune diseases, the interactions between LECs and T lymphocytes also regulate the immune microenvironment through various mechanisms. For example, in multiple sclerosis (MS), lymphatic vessels first transport CNS antigens (such as the myelin protein MOG) and DCs to deep cervical LNs, activating naïve T cells. Subsequently, autoreactive T cells acquire a specific migratory phenotype, particularly expressing molecules like the α4β1 integrin, lymphocyte function-associated antigen-1 (LFA-1), and CCR6. These T cells then interact with meningeal lymphatic endothelial cells via adhesion factors and chemokine pathways, promoting their infiltration into the central nervous system and exacerbating demyelinating lesions [116, 117]. Within the meninges of MS patients, LECs also participate in the formation of TLOs. Molecules expressed by LECs, such as podoplanin, facilitate the construction of B cell germinal centers and T cell activation clusters [116]. Therefore, investigating the potential functional role of the meningeal lymphatic system in the generation of tertiary lymphoid organs would be highly meaningful.

Furthermore, under inflammatory conditions, LECs stimulated by cytokines such as IFN-γ and TNF-α can directly inhibit T cell proliferation by releasing functional co-stimulatory molecules like nitric oxide and indoleamine 2,3-dioxygenase [38, 39] (Fig. 1e). On the other hand, TNF-α-stimulated LECs can reduce the expression of CD86 on the surface of dendritic cells, thereby impairing their ability to further stimulate T cell proliferation [27]. These studies suggest that during immune responses, LECs may help suppress potential excessive immune reactions by interfering with T cell activation.

### LEC-T Cell interactions in the tumor microenvironment

Within the tumor microenvironment, LECs interact with T cells through various mechanisms, forming a dynamic immunoregulatory network. These interactions encompass both negative regulation that promotes tumor immune escape and positive stimulation that enhances anti-tumor immunity, constituting a complex and balanced system [81, 118].

Clinical studies have revealed that in various solid tumors, T cells, particularly CD8^+^ T cells, are often “excluded” to the tumor periphery rather than infiltrating the core, forming so-called “immune-excluded” or “immune-desert” phenotypes [119, 120]. This phenomenon is closely associated with lymphatic vessel-mediated spatial redistribution of T cells [99, 121]. Studies have found that tumor-associated lymphatic endothelial cells upregulate the secretion of the chemokine CXCL12. By binding to the CXCR4 receptor on the T cell surface, CXCL12 actively guides T cells (especially CD8^+^ T cells expressing the stem-like marker TCF1^+^) to migrate along lymphatic vessels toward the tumor periphery [40] (Fig. 1f). Preclinical studies have confirmed that using CXCR4 inhibitors or genetically ablating CXCL12 in lymphatic endothelial cells can significantly enhance intratumoral T cell retention and improve the efficacy of immunotherapy in various cancers, including breast cancer, liver cancer, and melanoma [40, 122, 123].

A high frequency of tumor-infiltrating regulatory T cells (Tregs) has been observed in various human and murine solid tumors. These cells suppress anti-tumor immunity and are significantly associated with poor patient prognosis [124–126]. Compared to conventional CD4^+^ T cells, the hypoxic and acidic tumor microenvironment preferentially supports the survival and proliferation of Tregs [127]. Recent studies on human and mouse colon tumors have shown that constitutively CCR4-expressing Tregs preferentially accumulate in the peritumoral area, co-localizing with lymphatic vessels rather than blood vessels. It is plausible that migrating Tregs may directly interact with lymphatic endothelial cells (LECs), thereby promoting tumor growth by suppressing anti-tumor immunity [91]. Furthermore, among different T cell subsets, activated human and mouse Tregs express the highest levels of programmed death receptor-1 (PD-1) [41]. IFN-γ secreted by stromal cells in the TME can directly upregulate the expression of PD-L1 on tumor-associated lymphatic endothelial cells [111]. The PD-1 expressed on Tregs can selectively engage PD-L1 on LECs. This interaction transduces signals through the phosphoinositide 3-kinase (PI3K)-AKT and ERK signaling pathways, leading to downregulated expression of vascular endothelial cadherin (VE-cadherin) at LEC junctions and increased vascular permeability. This mechanism can also induce the activation of nuclear factor kappa B-p65 (NF-κB-p65) in LECs, which subsequently upregulates VCAM1 expression, promoting transendothelial migration (Fig. 1f). Pre-treatment with anti-PD-1 antibody reduced the capacity of intratumoral metastatic CD25^+^ Tregs to egress to dLNs, while simultaneously increasing their conversion rate to IFN-γ^+^CD25^−^ Tregs. These findings suggest that the PD-1/PD-L1 signaling pathway plays a regulatory role in the afferent migration of regulatory T cells to tumor-draining lymph nodes [41].

Additionally, LECs in the TME can directly suppress effector T cells. As mentioned, PD-L1 expression is upregulated on tumor-associated lymphatic endothelial cells in mouse melanoma and breast cancer models [111]. Immune co-localization studies show physical contact between T cells and PD-L1^+^ lymphatic vessels within tumors. The PD-L1 on LECs can directly inhibit CD8^+^ T cell activation (manifested as reduced CD25 and IFN-γ expression), significantly impairing the cytotoxic function of effector T cells against tumor cells [111]. This suggests that lymphatic PD-L1 could represent a novel target for overcoming resistance to immunotherapy.

Yet, it is important to note that the interactions between LECs and T cells in the tumor microenvironment are not solely limited to immunosuppressive negative functions. Research has found that in mouse melanoma or MC38 colorectal carcinoma models, in the presence of high densities of cytotoxic T lymphocytes (CTLs), IFN-γ secreted by CTLs enhances the tumor antigen cross-presentation capacity of tumor-associated LECs by upregulating genes such as NLRC5 and TAP1. This enables CTLs to directly induce apoptosis in LECs through MHC I-antigen-specific recognition, leading to a significant reduction in tumor lymphatic vessel density (LVD) and effectively inhibiting lymph node metastasis (Fig. 1f). This provides a critical theoretical basis for optimizing immunotherapies, such as adoptive T cell transfer, to block tumor dissemination [42]. A recent study also discovered that in melanoma, IFN-γ overexpressed by T cells regulates fatty acid metabolism in LECs by suppressing AMPK pathway activation. This, in turn, upregulates the expression of the tight junction protein Claudin-3, thereby enhancing lymphatic barrier function and inhibiting the transendothelial migration and lymphatic spread of melanoma cells. This finding offers new insights into the immunometabolic regulatory mechanisms of tumor metastasis [128].

Therefore, these findings suggest that tumor-associated LECs are not passive bystanders but active organizers of T-cell spatial distribution and functional fate. CXCL12-driven re-localization of TCF1⁺CD8⁺T cells to perilymphatic regions, PD-L1-mediated inhibition of effector T-cell activation, and preferential support of Treg egress to dLNs all converge to reinforce the immunosuppressive phenotype across multiple tumors. Meanwhile, high expression of IFN-γ in certain T cells induces a lymphatic endothelial cell cross-presentation mechanism coupled with upregulation of tight junction proteins, thereby reprogramming lymphatic vessels into effector structures that restrict metastatic spread. Rather than being inherently protumorigenic or antitumorigenic, LECs seem to adopt context-dependent states shaped by cytokine and metabolic cues. In the future, deciphering these states through single-cell and spatial multi-omics technologies and correlating them with clinical response patterns may elucidate the rationale for the synergistic effects of CXCR4 or PD-1/PD-L1-targeted therapies with pro-lymphangiogenesis therapies.

## Interactions between lymphatic endothelial cells and other immune cells

Besides T lymphocytes (80–90%) and dendritic cells (5–15%), which constitute the two predominant cellular populations in afferent lymph, other immune cells—such as B cells, neutrophils, and monocytes—are also present in afferent lymph, albeit in considerably smaller proportions [129, 130]. Therefore, this review consolidates the discussion of the interactions between these immune cells and LECs within this section.

### Lymphatic endothelial cells and B lymphocytes

**Fig. 2:**
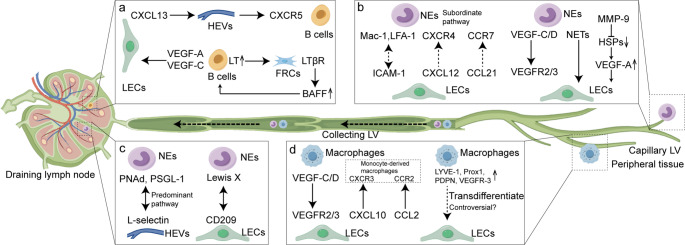
Interactions between lymphatic endothelial cells and B cells, neutrophils, and macrophages. (**a**) Lymphatic endothelial cells produce CXCL13 and transport it to the surface of HEVs, regulating B cell homing to lymph nodes via HEVs. Activated B cells express LT, activating LTβR signaling on FRCs and inducing BAFF secretion. BAFF synergizes with IL-4 to promote B cell production of vascular endothelial growth factors VEGF-A and VEGF-C, which directly stimulate LECs proliferation [131–134]. (**b**) Neutrophils migrate to draining lymph nodes via the lymphatic pathway primarily through integrin Mac-1 and LFA-1, as well as chemokine receptor CXCR4-mediated transendothelial migration. The classical CCL21-CCR7 axis may also contribute to the migration process. This lymphatic pathway represents a Subordinate pathway for neutrophil entry into lymph nodes. Neutrophils also promote lymphatic vessel formation by secreting VEGF-C/D and NETs, as well as by releasing VEGF-A bound to HSPs via MMP9 secretion [135–139]. (**c**) Neutrophil migration to lymph nodes via the HEVs pathway requires adhesion process mediated by L-selectin and PNAd/PSGL-1, which constitute the predominant pathway for neutrophil entry into lymph nodes [140]. Once neutrophils enter the lymph node sinuses, LECs express the C-type lectin CD209, which binds to the Lewis X sugar structure on neutrophils, mediating their adhesion and retention [13]. (**d**) Macrophages secrete VEGF-C and VEGF-D to stimulate LEC proliferation and migration via VEGFR3. Monocyte-derived macrophages migration into lymphatic vessels is guided by CXCL10 (binding to CXCR3 on macrophages) and the CCL2 gradient (shaped by LEC-expressed atypical chemokine receptor ACKR2 and sensed by macrophage CCR2). Under specific inflammatory or fibrotic conditions, macrophages may transdifferentiate into LECs and may integrate into the lymphatic vessel wall, contributing directly to lymphangiogenesis. Notably, this phenomenon and its mechanism remain controversial and context-dependent, and the current evidence is not sufficient to conclude stable in vivo lineage conversion [141–150]. Abbreviations:*LECs* Lymphatic endothelial cells, *CXCL13* C-X-C motif chemokine 13, *HEVs* high endothelial venules‌, *CXCR5* Chemokine C-X-C-motif receptor 5‌, *VEGF* vascular endothelial growth factor‌, *LT* lymphotoxin, *LTβR* lymphotoxin β receptor, *FRCs* fibroblastic reticular cells, *BAFF* B cell-activating factor, *NEs* Neutrophils, *Mac-1* Macrophage antigen-1, *LFA-1* Lymphocyte Function-associated Antigen-1, *ICAM-1* Intercellular adhesion molecule 1‌, *CXCR4* chemokine (C-X-C motif) receptor 4, *CXCL12* Chemokine (C-X-C motif) ligand 12, *CCR7* Chemokine (C-C motif) receptor 7, *CCL21* chemokine (C-C motif) ligand 21, *VEGF-R* vascular endothelial growth factor receptor‌, *NETs* neutrophil extracellular traps, *MMP-9* matrix metalloproteinase-9, *HSPs* heparan sulfate proteoglycans, *PNAd* Peripheral lymph node addressin, *PSGL-1* P-selectin glycoprotein ligand-1, *CXCR3* chemokine (C-X-C motif) receptor 3, *CXCL10* Chemokine (C-X-C motif) ligand 10, *CCR2* Chemokine (C-C motif) receptor 2, *CCL2* chemokine (C-C motif) ligand 2

Unlike T cells, circulating B cells highly express CXCR5 and are primarily guided by the chemokine CXCL13 to home to LNs via high endothelial venules (HEVs) [131]. Interestingly, CXCL13 is not directly produced by HEVs but depends on the expression of the non-canonical NF-κB-inducing kinase (NIK) in LECs. NIK induces CXCL13 expression, which is then transported to the surface of HEVs, directly regulating B cell homing to LNs and thereby influencing humoral immune responses [132] (Fig. 2a).

B cells also participate in the regulation and remodeling of lymphatic vessel architecture. Studies have shown that they can directly promote lymphangiogenesis through the expression of vascular endothelial growth factor (VEGF) [133]. In chronic inflammation, such as during infection with the intestinal helminth *Heligmosomoides polygyrus*, B cells, in concert with fibroblastic reticular cells (FRCs), drive lymphangiogenesis in the mesenteric lymph node via lymphotoxin beta receptor (LTβR)-dependent interactions. Activated B cells express lymphotoxin, which activates LTβR signaling on FRCs, inducing them to secrete B cell-activating factor (BAFF). BAFF, in synergy with interleukin-4 (IL-4), promotes both the production of VEGF-A and VEGF-C by B cells to directly stimulate LEC proliferation and enhances lymphotoxin expression in B cells, forming a positive feedback loop that expands the lymphatic network to sustain immune responses [134] (Fig. 2a). Interestingly, the influence of B cells on LECs can also impact the lymphatic homing of other immune cells. For instance, during helminth infection, the endogenous IL-4 receptor alpha chain (IL-4Rα) in B cells drives the expression of CCL24 in LECs, thereby recruiting eosinophils for drainage into the mesenteric LNs [151].

B cells are one of the major cellular constituents involved in the formation of TLOs during chronic inflammation [152, 153]. Three-dimensional imaging analysis of mesenteric tissues from Crohn’s disease patients has revealed the structural remodeling of collecting lymphatic vessels by B cell-enriched TLOs. In CD inflammatory regions, TLOs distribute along and invade the walls of collecting lymphatic vessels that drain toward the mesenteric LNs. Within these structures, B cells directly infiltrate the smooth muscle layer of the vessel wall, leading to a loss of wall integrity and impaired valve function. This provides crucial anatomical evidence for direct pathological interactions between lymphatic endothelium and B cells [154]. Recent work in the *TNF*^*ΔARE*^ mouse model of Crohn’s-like ileitis has revealed that distinct B cell lymphotoxin programs play non-redundant roles in coordinating TLOs formation and disease severity. B cell-derived membrane LTα1β2 is essential for the development of ileitis-associated TLOs along mesenteric collecting lymphatics, whereas soluble LTα3 produced by B cells counterbalances TNF-driven tissue damage [155]. Certainly, the molecular mechanisms and functional consequences of this interplay require further in-depth exploration. Future studies integrating single-cell sequencing and functional experiments are needed to decipher the code of this immune-lymphatic cross-talk.

### Lymphatic endothelial cells and neutrophils

Neutrophils (NEs) are crucial effector cells of the innate immune system. Under homeostatic conditions, circulating neutrophils continuously patrol the body’s draining lymph nodes [156]. They primarily enter LNs via high endothelial venules in an L-selectin-dependent manner and localize to the nodal interstitium; their egress from LNs is then mediated by sphingosine-1-phosphate receptors through the efferent lymphatic vessels [157].

Upon the initiation of an inflammatory response (e.g., infection, tissue injury), neutrophils rapidly migrate to the site of damage. They eliminate pathogens and promote immune cell recruitment through phagocytosis, intracellular degradation, granule release, and the formation of extracellular traps [158]. While the majority of neutrophils that enter the inflammatory tissue die at the injury site, a subset can re-enter the bloodstream via reverse migration and subsequently re-infiltrate dLNs from the circulation through high endothelial venules [140, 159, 160]. Importantly, direct in vivo tracking in a peripheral Staphylococcus aureus infection model showed that the rapid neutrophil influx into the lymph node is largely driven by PNAd/L-selectin-dependent recruitment through HEVs, supporting HEV entry as a predominant route in this setting [140] (Fig. 2c).

Neutrophils have also been detected within afferent lymph and inside inflamed lymphatic vessels in several experimental infection/inflammation models, leading to the proposal that a smaller fraction may access dLNs via afferent lymphatics [135, 136]. However, in many of these studies neutrophils are observed in dLNs only after strong inflammatory stimulation and alternative routes of entry (notably HEVs) cannot always be fully excluded; thus, evidence for constitutive or general neutrophil trafficking from tissues to lymph nodes via afferent lymphatics remains limited and likely context-dependent. When lymphatic entry has been observed, it has been reported to involve integrins Mac-1 and LFA-1 and CXCR4-mediated transendothelial migration across inflamed lymphatic endothelium [135, 137]. Notably, neutrophils have been described to transit inflamed lymphatic endothelium via integrin-dependent proteolysis and junctional retraction, creating larger migration channels rather than relying exclusively on pre-existing endothelial junctions [161]. The classical CCL21-CCR7 axis may also participate in neutrophil migration to LNs. Studies have shown that TNF-α coordinates neutrophil crawling along afferent lymphatic vessels toward a CCL21 concentration gradient in a CCR7-dependent manner [162]. In contrast, other models (e.g., Staphylococcus aureus-induced skin inflammation) support CCR7-independent migration requirements (CXCR4 and CD11b) [135] (Fig. 2b), underscoring that distinct stimuli and phases of inflammation may engage different molecular programs. By comparison, HEV recruitment additionally requires the initial adhesion and rolling steps mediated by L-selectin and P-selectin glycoprotein ligand-1 (PSGL-1) and can depend on PNAd [137, 140] (Fig. 2c). Together, current evidence suggests that neutrophil entry into lymph nodes is dominated by HEVs in homeostasis and in several acute infection models, whereas afferent lymphatic migration represents a less common, context-dependent pathway that should not be overgeneralized. Clarifying when CCR7 is a general versus context-restricted regulator of neutrophil–LEC interactions will be important before pursuing it as a therapeutic target.

LECs can also mediate the retention of neutrophils within LNs. Single-cell sequencing analysis of lymphatic vessels revealed that LECs in the lymph node medullary sinus specifically bind to the Lewis X (CD15) glycan structure on neutrophils via the surface C-type lectin CD209. This interaction mediates the directed adhesion of neutrophils in the medullary sinus, forming an immune barrier that prevents the dissemination of lymph-borne pathogens [13] (Fig. 2c). This novel mechanism provides a new direction for enhancing anti-infection defense by targeting lymphatic vessels.

Neutrophils also promote lymphangiogenesis in inflammatory and cancerous contexts through multiple pathways. Firstly, neutrophils directly secrete VEGF-C and VEGF-D, which activate the VEGFR2/3 signaling pathway on LECs, promoting lymphatic endothelial cell proliferation, migration, and tube formation [138, 163, 164]. Concurrently, by secreting matrix metalloproteinase-9 (MMP9) and heparinase, neutrophils degrade heparan sulfate proteoglycans (HSPs) in the extracellular matrix, thereby releasing bound VEGF-A and significantly enhancing its bioavailability [138] (Fig. 2b). Furthermore, neutrophil extracellular traps (NETs)—complex web-like structures composed of DNA-histone complexes and proteins released by neutrophils—have been well-studied for their role in angiogenesis [165, 166]. Recent research has focused on the relationship between NETs and lymphangiogenesis. During lymphatic metastasis in cervical cancer, NETs produced by infiltrating neutrophils directly stimulate lymphatic endothelial cells, significantly increasing lymphatic vessel length and branching, and disrupting lymphatic endothelial barrier integrity. These effects collectively promote the metastasis of cancer cells to LNs via the lymphatic system [139] (Fig. 2b). However, current studies have primarily emphasized the functional impact of NETs on LECs; the specific underlying molecular signaling mechanisms still require further exploration.

### Lymphatic endothelial cells and macrophages

Macrophages express a variety of cytokines, including VEGF-A, VEGF-B, VEGF-C, VEGF-D, and PLGF from the VEGF family, which are closely associated with lymphangiogenesis [167–169]. Notably, during development, macrophages are confirmed to be the primary source of lymphangiogenic growth factors required for developmental lymphangiogenesis. Studies on macrophage colony-stimulating factor (M-CSF)-deficient (op/op) mice revealed that when macrophage abundance is reduced, these op/op mice exhibit delayed postnatal lymphatic development [170]. Research by Böhmer et al. also demonstrated that macrophages expressing the cytoplasmic tyrosine kinase Syk directly drive the proliferation and migration of lymphatic endothelial cells by secreting VEGF-C and VEGF-D, thereby regulating developmental lymphangiogenesis [141] (Fig. 2d).

In adulthood, interactions between macrophages and lymphatic vessels primarily occur during tissue injury and inflammatory responses. Following inflammatory damage, bone marrow–derived myeloid cells, including inflammatory monocytes and monocyte-derived macrophages, are recruited to the injured tissue. These cells contribute to inflammation resolution by phagocytosing antigens and clearing cellular debris, thereby facilitating antigen clearance and restoration of tissue homeostasis [171]. In an acute skin inflammation setting, macrophage accumulation in draining lymph nodes (dLNs) was reported to be strongly influenced by enhanced lymphatic function, and the authors proposed that draining lymphatics may represent a major route by which monocyte-derived macrophages access dLNs [171]. However, this conclusion was largely inferred from tissue localization and functional perturbation of lymphatic function, rather than from direct intralymphatic tracing or real-time imaging demonstrating macrophage entry into lymphatic lumens and transport to dLNs. Although myeloid cells can be detected in afferent lymph and in draining lymph nodes after inflammatory challenge, direct evidence that mature tissue-resident macrophages actively traffic from tissues to dLNs via afferent lymphatic vessels remains limited. Instead, monocytic precursors have been shown to enter lymphatic vessels from the dermis and reach dLNs in a CCR7-dependent manner in some settings [142]. Accordingly, the molecular mechanisms that regulate recruitment of monocytes/monocyte-derived macrophages to lymphatic vessels and their subsequent fate remain incompletely understood. Studies using human monocytic cells (THP-1) or peritoneal monocyte-derived cells reported that TNF-α–stimulated LECs secrete CXCL10 and that LPS can induce LEC Toll-like receptor 4 (TLR4) expression, together promoting recruitment/adhesion of CXCR3⁺ monocytes/monocyte-derived macrophages to draining lymphatic vessels [143, 144]. Furthermore, the atypical chemokine receptor 2 (ACKR2) expressed on LECs influences the distance between CCR2-positive myeloid cells and the lymphatic vessel wall by shaping the concentration gradient of CCL2 secreted by LECs [172] (Fig. 2d).

Naturally, during inflammatory injury, the regulation between macrophages and lymphatic endothelial cells is bidirectional. Macrophage-derived lymphangiogenic growth factors can participate in lymphatic remodeling through paracrine signaling pathways. In a liver ischemia/reperfusion injury model, infiltrating macrophages promoted lymphangiogenesis in the injured area by secreting VEGF-C/D, which activates the VEGFR3 signaling pathway on lymphatic endothelial cells. This enhances tissue fluid drainage function and accelerates the clearance of necrotic tissue and the restoration of microenvironmental homeostasis [173]. Studies related to lymphedema have also found that the lymphedema microenvironment induces macrophage polarization towards a pro-lymphangiogenic M2 phenotype. In turn, VEGF-C secreted by these macrophages directly supports the survival and function of lymphatic endothelial cells, forming a positive feedback loop for inflammation-repair [174].

Beyond their paracrine roles in development and inflammation, some studies have reported macrophage-lineage or mesenchymal cells near developing or inflamed lymphatic vessels that co-express lymphatic-associated markers, raising the possibility that these cells may contribute to lymphangiogenesis through cell-autonomous mechanisms [145, 146, 175]. The prevailing view holds that LECs originate from cardinal vein endothelial cells [147]. During lymphatic development, CD45⁺, F4/80⁺ cells co-expressing LYVE-1 and/or Prox-1 have been observed in close proximity to growing lymphatic vessels [145]. However, several “lymphatic markers” (e.g., LYVE-1, podoplanin) can also be expressed by tissue-resident macrophages and other stromal cells, and marker co-expression alone does not establish lineage conversion into functional LECs. In vitro, LPS-treated RAW264.7 macrophage-like cells upregulated VEGFR-3 transcripts and surface protein and other lymphatic-associated programs, consistent with inducible acquisition of a lymphatic-like phenotype under inflammatory cues, but this does not by itself demonstrate stable transdifferentiation into bona fide LECs [146] (Fig. 2d).

Additional reports in fibrotic or tumor microenvironments have described macrophages that upregulate lymphatic-associated markers, and can form lymphatic-like structures in co-culture systems. These observations have been interpreted by some as potential macrophage-to-LEC transdifferentiation [148–150, 176]. For example, in renal fibrosis, aldosterone–mineralocorticoid receptor (MR) signaling was reported to increase macrophage VEGF-C production and to induce expression of LEC-associated markers, an effect inhibited by the MR blocker esaxerenone [148]. This transdifferentiation phenomenon is even more common in tumor environments [149, 150]. In tumor settings, F4/80⁺CD11b⁺ macrophages co-cultured with the 4T1 breast cancer cell line increased expression of LYVE-1, Prox1, podoplanin, and VEGFR-3 and formed clusters reminiscent of lymphatic-like structures in vitro [176] (Fig. 2d). Certainly, the notion that macrophages can transdifferentiate into bona fide LECs and structurally integrate into lymphatic vessels remains highly debated. Many of the supporting studies rely primarily on marker expression or co-expression (e.g., LYVE-1, Prox1, podoplanin, VEGFR-3) in vitro or in inflamed/fibrotic tissues. Importantly, several of these “lymphatic” markers can also be detected in LYVE-1⁺ tissue-resident macrophages and other stromal populations, and therefore marker positivity alone does not establish stable lineage conversion into functional LECs in vivo [149]. Such findings may instead reflect transient acquisition of a lymphangiogenic/LEC-like phenotype under inflammatory cues rather than durable endothelial identity. Moreover, rigorous genetic lineage tracing at single-cell resolution remains sparse, and the long-term functional contribution of putative macrophage-derived LEC-like cells to lymphatic vessel integrity, lymph transport, and immune cell trafficking is largely unknown. Thus, while macrophage plasticity clearly contributes to lymphangiogenesis through growth-factor secretion and may be accompanied by cell-autonomous adoption of LEC-associated programs in certain contexts, the field has not yet reached consensus on whether macrophages should be regarded as true LEC progenitors in vivo. Addressing this question will require stringent genetic fate-mapping combined with functional assays that directly test incorporation into lymphatic endothelium and its impact on vessel structure and transport.

With the widespread application of single-cell sequencing technology, the intrinsic heterogeneity of LECs themselves is increasingly recognized as a key determinant of immunomodulatory function. A striking recent example of such LEC specialization is a PTX3-expressing subtype at lymphatic capillary terminals. Single-cell and functional studies have identified these PTX3⁺ terminal LECs as “immune-interacting” cells that recruit pro-lymphangiogenic macrophages and control terminal lymphatic branching and valve-like structures, shaping the lymphatic structure and immune microenvironment [177]. Such discoveries of defined LEC subtypes with specific programs move the field toward a precise understanding of how molecularly distinct LECs execute immune modulation [178, 179].

## Conclusion and Future perspectives

In summary, we can obtain several common mechanisms of the interaction between LECs and immune cells. LECs, as architects of chemokine gradient landscapes, form spatial niches and “decision points” with the structural features of lymphatic vessels, regulating the entry, migration, and lymph node homing of various immune cell subsets. Simultaneously, LECs serve as context-dependent immunoregulatory hubs: in steady state they promote tolerance via self-antigen archiving and Treg support, whereas during acute inflammation they are rapidly reprogrammed by cytokines and growth factors to enhance antigen transport and effector cell recruitment, and under chronic conditions the same programs can drive tertiary lymphoid structures, autoimmunity or immunosuppressive tumor microenvironments. Thus, a limited set of LEC modules—chemokines, co-stimulatory/co-inhibitory ligands, antigen-presentation machinery and S1P gradients—is reused in different combinations to coordinate DCs, T cells, B cells, neutrophils and macrophages in a highly context-dependent manner.

From a translational standpoint, the mechanisms summarized here point to several therapeutic strategies targeting LEC–immune crosstalk. In cancer, blocking VEGF-C/VEGFR3-driven lymphangiogenesis or CCR7–CCL21-dependent trafficking may limit lymph node metastasis and reshape antitumor immunity, whereas selectively inhibiting PD-L1 on LECs could relieve lymphatic checkpoints that delete tumor-specific CD8⁺ T cells. Conversely, in chronic inflammatory disease and secondary lymphedema, VEGF-C–based gene or mRNA therapies may restore lymphatic drainage and promote resolution. Defining when to inhibit versus promote lymphatic growth, and how to combine such approaches with immunotherapies, will be crucial for precision interventions.

Although significant progress has been made in understanding LEC-immune cell interactions, key questions remain. First, recent single-cell and spatial multi-omics studies have revealed substantial heterogeneity among LECs across lymph node sinuses and peripheral organs, but the organ- and niche-specific immunoregulatory roles of these subsets remain poorly defined and need to be addressed using advanced in vivo imaging and spatiotemporal omics. Second, more precise therapeutic strategies targeting this crosstalk are required, such as combining existing immunotherapies with the modulation of immunosuppressive tumor-associated LECs. Third, future studies should shift focus from immune cell receptors to LEC-specific targets (e.g., chemokine expression) to develop more precise interventions with fewer side effects. Fourth, lineage tracing and single-cell multi-omics are crucial for resolving fate transitions, such as macrophage-to-LEC transdifferentiation, and assessing the stability and microenvironmental regulation of these processes. Finally, strengthening humanized models, patient-derived single-cell analyses, and organoid systems will be vital for validating findings and accelerating clinical translation. Integrating these multidisciplinary approaches will be essential to decipher the spatiotemporal dynamics of lymphatic-immune crosstalk and ultimately achieve disease treatment by remodeling the lymphatic microenvironment.

## Data Availability

No data was generated as part of this paper.

## References

[1] Davis MJ, Zawieja SD, King PD (2025) Transport and immune functions of the lymphatic system. Annu Rev Physiol 87(1):151–72. 10.1146/annurev-physiol-022724-104908 39441893 10.1146/annurev-physiol-022724-104908

[2] Roth KS, Pabst R (2025) The functional unit of the lymphatic system: towards understanding the importance of a well-rehearsed interaction of lymphatic capillaries, collecting vessels, and lymph nodes. J Vasc Res 62(4):183–92. 10.1159/000545084 40101694 10.1159/000545084

[3] Baluk P, Mcdonald DM (2022) Buttons and zippers: endothelial junctions in lymphatic vessels. Cold Spring Harb Perspect Med. 10.1101/cshperspect.a041178 35534209 10.1101/cshperspect.a041178 PMC9643678

[4] Baluk P, Fuxe J, Hashizume H et al (2007) Functionally specialized junctions between endothelial cells of lymphatic vessels. J Exp Med 204(10):2349–62. 10.1084/jem.20062596 17846148 10.1084/jem.20062596 PMC2118470

[5] Schoofs H, Daubel N, Schnabellehner S et al (2025) Dynamic cytoskeletal regulation of cell shape supports resilience of lymphatic endothelium. Nature 641(8062):465–75. 10.1038/s41586-025-08724-6 40108458 10.1038/s41586-025-08724-6 PMC12058511

[6] Bauer A, Tatliadim H, Halin C (2022) Leukocyte trafficking in lymphatic vessels. Cold Spring Harb Perspect Med. 10.1101/cshperspect.a041186 35379657 10.1101/cshperspect.a041186 PMC9524389

[7] Card CM, Yu SS, Swartz MA (2014) Emerging roles of lymphatic endothelium in regulating adaptive immunity. J Clin Invest 124(3):943–52. 10.1172/jci73316 24590280 10.1172/JCI73316 PMC3938271

[8] Hampton HR, Chtanova T (2019) Lymphatic migration of immune cells. Front Immunol 10:1168. 10.3389/fimmu.2019.01168 31191539 10.3389/fimmu.2019.01168 PMC6546724

[9] Santambrogio L, Berendam SJ, Engelhard VH (2019) The antigen processing and presentation machinery in lymphatic endothelial cells. Front Immunol 10:1033. 10.3389/fimmu.2019.01033 31134089 10.3389/fimmu.2019.01033 PMC6513971

[10] Podgrabinska S, Skobe M (2014) Role of lymphatic vasculature in regional and distant metastases. Microvasc Res 95:46–52. 10.1016/j.mvr.2014.07.004 25026412 10.1016/j.mvr.2014.07.004 PMC4446725

[11] Padera TP, Kadambi A, DI Tomaso E et al (2002) Lymphatic metastasis in the absence of functional intratumor lymphatics. Science 296(5574):1883–6. 10.1126/science.1071420 11976409 10.1126/science.1071420

[12] Qian CN, Berghuis B, Tsarfaty G et al (2006) Preparing the “soil”: the primary tumor induces vasculature reorganization in the sentinel lymph node before the arrival of metastatic cancer cells. Cancer Res 66(21):10365–76. 10.1158/0008-5472.Can-06-2977 17062557 10.1158/0008-5472.CAN-06-2977

[13] Takeda A, HOLLMéN M, Dermadi D et al (2019) Single-cell survey of human lymphatics unveils marked endothelial cell heterogeneity and mechanisms of homing for neutrophils. Immunity 51(3):561–72.e5. 10.1016/j.immuni.2019.06.027 31402260 10.1016/j.immuni.2019.06.027

[14] Fujimoto N, He Y, D’addio M et al (2020) Single-cell mapping reveals new markers and functions of lymphatic endothelial cells in lymph nodes. PLoS Biol 18(4):e3000704. 10.1371/journal.pbio.3000704 32251437 10.1371/journal.pbio.3000704 PMC7162550

[15] Miyasaka M (2021) A short review on lymphatic endothelial cell heterogeneity. Inflamm Regen 41(1):32. 10.1186/s41232-021-00183-6 34635187 10.1186/s41232-021-00183-6 PMC8507377

[16] Randolph GJ, Beaulieu S, Pope M et al (1998) A physiologic function for p-glycoprotein (MDR-1) during the migration of dendritic cells from skin via afferent lymphatic vessels. Proc Natl Acad Sci U S A 95(12):6924–9. 10.1073/pnas.95.12.6924 9618515 10.1073/pnas.95.12.6924 PMC22688

[17] Randolph GJ, Angeli V, Swartz MA (2005) Dendritic-cell trafficking to lymph nodes through lymphatic vessels. Nat Rev Immunol 5(8):617–28. 10.1038/nri1670 16056255 10.1038/nri1670

[18] Weber M, Hauschild R, Schwarz J et al (2013) Interstitial dendritic cell guidance by haptotactic chemokine gradients. Science 339(6117):328–32. 10.1126/science.1228456 23329049 10.1126/science.1228456

[19] Britschgi MR, Favre S, Luther SA (2010) CCL21 is sufficient to mediate DC migration, maturation and function in the absence of CCL19. Eur J Immunol 40(5):1266–71. 10.1002/eji.200939921 20201039 10.1002/eji.200939921

[20] Holtkamp SJ, Ince LM, Barnoud C et al (2021) Circadian clocks guide dendritic cells into skin lymphatics. Nat Immunol 22(11):1375–81. 10.1038/s41590-021-01040-x 34663979 10.1038/s41590-021-01040-x PMC8553624

[21] Rathinasamy A, Czeloth N, Pabst O et al (2010) The origin and maturity of dendritic cells determine the pattern of sphingosine 1-phosphate receptors expressed and required for efficient migration. Journal of immunology (Baltimore, Md : 1950) 185(7):4072–81. 10.4049/jimmunol.1000568 20826749 10.4049/jimmunol.1000568

[22] Acton SE, Astarita JL, Malhotra D et al (2012) Podoplanin-rich stromal networks induce dendritic cell motility via activation of the C-type lectin receptor CLEC-2. Immunity 37(2):276–89. 10.1016/j.immuni.2012.05.022 22884313 10.1016/j.immuni.2012.05.022 PMC3556784

[23] Haghayegh Jahromi N, Gkountidi AO, Collado-Diaz V et al (2024) CD112 supports lymphatic migration of human dermal dendritic cells. Cells. 10.3390/cells13050424 38474388 10.3390/cells13050424 PMC10931060

[24] Teijeira Á, PALAZóN A, Garasa S et al (2012) CD137 on inflamed lymphatic endothelial cells enhances CCL21-guided migration of dendritic cells. FASEB J 26(8):3380–92. 10.1096/fj.11-201061 22593548 10.1096/fj.11-201061

[25] Chen KY, DE Giovanni M, Xu Y et al (2025) Inflammation switches the chemoattractant requirements for naive lymphocyte entry into lymph nodes. Cell 188(4):1019–35.e22. 10.1016/j.cell.2024.11.031 39708807 10.1016/j.cell.2024.11.031 PMC11845304

[26] Betterman KL, Harvey NL (2016) The lymphatic vasculature: development and role in shaping immunity. Immunol Rev 271(1):276–92. 10.1111/imr.12413 27088921 10.1111/imr.12413

[27] Podgrabinska S, Kamalu O, Mayer L et al (2009) Inflamed lymphatic endothelium suppresses dendritic cell maturation and function via Mac-1/ICAM-1-dependent mechanism. J Immunol 183(3):1767–79. 10.4049/jimmunol.0802167 19587009 10.4049/jimmunol.0802167 PMC4410990

[28] Scandella E, Men Y, Gillessen S et al (2002) Prostaglandin E2 is a key factor for CCR7 surface expression and migration of monocyte-derived dendritic cells. Blood 100(4):1354–61. 10.1182/blood-2001-11-0017 12149218 10.1182/blood-2001-11-0017

[29] Swartz MA (2014) Immunomodulatory roles of lymphatic vessels in cancer progression. Cancer Immunol Res 2(8):701–7. 10.1158/2326-6066.Cir-14-0115 25092811 10.1158/2326-6066.CIR-14-0115

[30] Maier B, Leader AM, Chen ST et al (2020) A conserved dendritic-cell regulatory program limits antitumour immunity. Nature 580(7802):257–62. 10.1038/s41586-020-2134-y 32269339 10.1038/s41586-020-2134-y PMC7787191

[31] Bromley SK, Thomas SY, Luster AD (2005) Chemokine receptor CCR7 guides T cell exit from peripheral tissues and entry into afferent lymphatics. Nat Immunol 6(9):895–901. 10.1038/ni1240 16116469 10.1038/ni1240

[32] Mackay LK, Braun A, Macleod BL et al (2015) Cutting edge: CD69 interference with sphingosine-1-phosphate receptor function regulates peripheral T cell retention. Journal of immunology (Baltimore, Md : 1950) 194(5):2059–63. 10.4049/jimmunol.1402256 25624457 10.4049/jimmunol.1402256

[33] Marttila-Ichihara F, Turja R, Miiluniemi M et al (2008) Macrophage mannose receptor on lymphatics controls cell trafficking. Blood 112(1):64–72. 10.1182/blood-2007-10-118984 18434610 10.1182/blood-2007-10-118984

[34] Brinkman CC, Iwami D, Hritzo MK et al (2016) Treg engage lymphotoxin beta receptor for afferent lymphatic transendothelial migration. Nature communications 7:12021. 10.1038/ncomms12021 27323847 10.1038/ncomms12021 PMC4919545

[35] Johnson LA, Jackson DG (2010) Inflammation-induced secretion of CCL21 in lymphatic endothelium is a key regulator of integrin-mediated dendritic cell transmigration. Int Immunol 22(10):839–49. 10.1093/intimm/dxq435 20739459 10.1093/intimm/dxq435

[36] Watanabe R, Gehad A, Yang C et al (2015) Human skin is protected by four functionally and phenotypically discrete populations of resident and recirculating memory T cells. Sci Transl Med 7(279):279ra39. 10.1126/scitranslmed.3010302 25787765 10.1126/scitranslmed.3010302 PMC4425193

[37] Teijeira A, Hunter MC, Russo E et al (2017) T cell migration from inflamed skin to draining lymph nodes requires intralymphatic crawling supported by ICAM-1/LFA-1 interactions. Cell Rep 18(4):857–65. 10.1016/j.celrep.2016.12.078 28122237 10.1016/j.celrep.2016.12.078

[38] NöRDER M, Gutierrez MG, Zicari S et al (2012) Lymph node-derived lymphatic endothelial cells express functional costimulatory molecules and impair dendritic cell-induced allogenic T-cell proliferation. FASEB J 26(7):2835–46. 10.1096/fj.12-205278 22459150 10.1096/fj.12-205278

[39] Lukacs-Kornek V, Malhotra D, Fletcher AL et al (2011) Regulated release of nitric oxide by nonhematopoietic stroma controls expansion of the activated T cell pool in lymph nodes. Nat Immunol 12(11):1096–104. 10.1038/ni.2112 21926986 10.1038/ni.2112 PMC3457791

[40] Steele MM, Jaiswal A, Delclaux I et al (2023) T cell egress via lymphatic vessels is tuned by antigen encounter and limits tumor control. Nat Immunol 24(4):664–75. 10.1038/s41590-023-01443-y 36849745 10.1038/s41590-023-01443-y PMC10998279

[41] Piao W, Li L, Saxena V et al (2022) PD-L1 signaling selectively regulates T cell lymphatic transendothelial migration. Nat Commun 13(1):2176. 10.1038/s41467-022-29930-0 35449134 10.1038/s41467-022-29930-0 PMC9023578

[42] Garnier L, Pick R, Montorfani J et al (2022) IFN-γ-dependent tumor-antigen cross-presentation by lymphatic endothelial cells promotes their killing by T cells and inhibits metastasis. Sci Adv 8(23):eabl5162. 10.1126/sciadv.abl5162 35675399 10.1126/sciadv.abl5162 PMC9176743

[43] Friess MC, Kritikos I, Schineis P et al (2022) Mechanosensitive ACKR4 scavenges CCR7 chemokines to facilitate T cell de-adhesion and passive transport by flow in inflamed afferent lymphatics. Cell Rep 38(5):110334. 10.1016/j.celrep.2022.110334 35108538 10.1016/j.celrep.2022.110334

[44] Johnson LA, Banerji S, Lawrance W et al (2017) Dendritic cells enter lymph vessels by hyaluronan-mediated docking to the endothelial receptor LYVE-1. Nat Immunol 18(7):762–70. 10.1038/ni.3750 28504698 10.1038/ni.3750

[45] Johnson LA, Clasper S, Holt AP et al (2006) An inflammation-induced mechanism for leukocyte transmigration across lymphatic vessel endothelium. J Exp Med 203(12):2763–77. 10.1084/jem.20051759 17116732 10.1084/jem.20051759 PMC2118156

[46] Vicente-Manzanares M, Ma X, Adelstein RS et al (2009) Non-muscle myosin II takes centre stage in cell adhesion and migration. Nat Rev Mol Cell Biol 10(11):778–90. 10.1038/nrm2786 19851336 10.1038/nrm2786 PMC2834236

[47] Nourshargh S, Hordijk PL, Sixt M (2010) Breaching multiple barriers: leukocyte motility through venular walls and the interstitium. Nat Rev Mol Cell Biol 11(5):366–78. 10.1038/nrm2889 20414258 10.1038/nrm2889

[48] Smith A, Bracke M, Leitinger B et al (2003) LFA-1-induced T cell migration on ICAM-1 involves regulation of MLCK-mediated attachment and ROCK-dependent detachment. J Cell Sci 116(Pt 15):3123–33. 10.1242/jcs.00606 12799414 10.1242/jcs.00606

[49] NITSCHKé M, Aebischer D, Abadier M et al (2012) Differential requirement for ROCK in dendritic cell migration within lymphatic capillaries in steady-state and inflammation. Blood 120(11):2249–58. 10.1182/blood-2012-03-417923 22855606 10.1182/blood-2012-03-417923

[50] Vigl B, Aebischer D, NITSCHKé M et al (2011) Tissue inflammation modulates gene expression of lymphatic endothelial cells and dendritic cell migration in a stimulus-dependent manner. Blood 118(1):205–15. 10.1182/blood-2010-12-326447 21596851 10.1182/blood-2010-12-326447

[51] Alvarez D, Vollmann EH, VON Andrian UH (2008) Mechanisms and consequences of dendritic cell migration. Immunity 29(3):325–42. 10.1016/j.immuni.2008.08.006 18799141 10.1016/j.immuni.2008.08.006 PMC2818978

[52] MARTíN-FONTECHA A, LANZAVECCHIA A, SALLUSTO F (2009) Dendritic cell migration to peripheral lymph nodes [J]. Handb Exp Pharmacol 18831–49. 10.1007/978-3-540-71029-5_2 10.1007/978-3-540-71029-5_2 19031020

[53] Ivanov S, Scallan JP, Kim KW et al (2016) CCR7 and IRF4-dependent dendritic cells regulate lymphatic collecting vessel permeability. J Clin Invest 126(4):1581–91. 10.1172/jci84518 26999610 10.1172/JCI84518 PMC4811132

[54] FöRSTER R, Schubel A, Breitfeld D et al (1999) CCR7 coordinates the primary immune response by establishing functional microenvironments in secondary lymphoid organs. Cell 99(1):23–33. 10.1016/s0092-8674(00)80059-8 10520991 10.1016/s0092-8674(00)80059-8

[55] Liu J, Cheng Y, Zhang X et al (2023) Glycosyltransferase Extl1 promotes CCR7-mediated dendritic cell migration to restrain infection and autoimmunity. Cell Rep 42(1):111991. 10.1016/j.celrep.2023.111991 36656709 10.1016/j.celrep.2023.111991

[56] Ulvmar MH, Werth K, Braun A et al (2014) The atypical chemokine receptor CCRL1 shapes functional CCL21 gradients in lymph nodes. Nat Immunol 15(7):623–30. 10.1038/ni.2889 24813163 10.1038/ni.2889

[57] Braun A, Worbs T, Moschovakis GL et al (2011) Afferent lymph-derived T cells and DCs use different chemokine receptor CCR7-dependent routes for entry into the lymph node and intranodal migration. Nat Immunol 12(9):879–87. 10.1038/ni.2085 21841786 10.1038/ni.2085

[58] Johnson LA, Jackson DG (2013) The chemokine CX3CL1 promotes trafficking of dendritic cells through inflamed lymphatics. J Cell Sci 126(Pt 22):5259–70. 10.1242/jcs.135343 24006262 10.1242/jcs.135343 PMC3828594

[59] CROMER W E, ZAWIEJA S D THARAKANB et al (2014) The effects of inflammatory cytokines on lymphatic endothelial barrier function [J]. Angiogenesis 17(2):395–406. 10.1007/s10456-013-9393-2 24141404 10.1007/s10456-013-9393-2 PMC4314095

[60] Zhou R, To KK, Wong YC et al (2020) Acute SARS-CoV-2 infection impairs dendritic cell and t cell responses. Immunity 53(4):864–77.e5. 10.1016/j.immuni.2020.07.026 32791036 10.1016/j.immuni.2020.07.026 PMC7402670

[61] Dhaiban S, Al-Ani M, Elemam NM et al (2020) Targeting chemokines and chemokine receptors in Multiple Sclerosis and Experimental Autoimmune Encephalomyelitis. J Inflamm Res 13:619–33. 10.2147/jir.S270872 33061527 10.2147/JIR.S270872 PMC7532903

[62] Miyabe Y, Lian J, Miyabe C et al (2019) Chemokines in rheumatic diseases: pathogenic role and therapeutic implications. Nat Rev Rheumatol 15(12):731–46. 10.1038/s41584-019-0323-6 31705045 10.1038/s41584-019-0323-6

[63] Zhang QY, Ye XP, Zhou Z et al (2022) Lymphocyte infiltration and thyrocyte destruction are driven by stromal and immune cell components in Hashimoto’s thyroiditis. Nat Commun 13(1):775. 10.1038/s41467-022-28120-2 35140214 10.1038/s41467-022-28120-2 PMC8828859

[64] CAMBA-GóMEZ M, Arosa L, Gualillo O et al (2022) Chemokines and chemokine receptors in inflammatory bowel disease: recent findings and future perspectives. Drug Discov Today 27(4):1167–75. 10.1016/j.drudis.2021.12.004 34896626 10.1016/j.drudis.2021.12.004

[65] Coutant F, Miossec P (2016) Altered dendritic cell functions in autoimmune diseases: distinct and overlapping profiles. Nat Rev Rheumatol 12(12):703–15. 10.1038/nrrheum.2016.147 27652503 10.1038/nrrheum.2016.147

[66] Han Y, Li X, Zhou Q et al (2015) FTY720 abrogates collagen-induced arthritis by hindering dendritic cell migration to local lymph nodes. J Immunol 195(9):4126–35. 10.4049/jimmunol.1401842 26416269 10.4049/jimmunol.1401842

[67] MOSCHOVAKIS GL, BUBKE A, FRIEDRICHSEN M et al (2019) The chemokine receptor CCR7 is a promising target for rheumatoid arthritis therapy [J]. Cell Mol Immunol 16(10):791–799. 10.1038/s41423-018-0056-5 29973648 10.1038/s41423-018-0056-5 PMC6804778

[68] Hong W, Ma H, Yang Z et al (2025) Optineurin restrains CCR7 degradation to guide type II collagen-stimulated dendritic cell migration in Rheumatoid Arthritis. Acta Pharm Sin B 15(3):1626–42. 10.1016/j.apsb.2025.02.004 40370566 10.1016/j.apsb.2025.02.004 PMC12069250

[69] Rahier JF, DE Beauce S, Dubuquoy L et al (2011) Increased lymphatic vessel density and lymphangiogenesis in Inflammatory Bowel Disease. Aliment Pharmacol Ther 34(5):533–43. 10.1111/j.1365-2036.2011.04759.x 21736598 10.1111/j.1365-2036.2011.04759.x

[70] Rehal S, Stephens M, Roizes S et al (2018) Acute small intestinal inflammation results in persistent lymphatic alterations. Am J Physiol Gastrointest Liver Physiol 314(3):G408-g17. 10.1152/ajpgi.00340.2017 29351397 10.1152/ajpgi.00340.2017

[71] Geleff S, Schoppmann SF, Oberhuber G (2003) Increase in podoplanin-expressing intestinal lymphatic vessels in Inflammatory Bowel Disease. Virchows Arch 442(3):231–7. 10.1007/s00428-002-0744-4 12647212 10.1007/s00428-002-0744-4

[72] Czepielewski RS, Erlich EC, Onufer EJ et al (2021) Ileitis-associated tertiary lymphoid organs arise at lymphatic valves and impede mesenteric lymph flow in response to tumor necrosis factor. Immunity 54(12):2795–811.e9. 10.1016/j.immuni.2021.10.003 34788601 10.1016/j.immuni.2021.10.003 PMC8678349

[73] Shu W, Wang Y, Li C et al (2023) Single-cell expression atlas reveals cell heterogeneity in the creeping fat of Crohn’s Disease. Inflamm Bowel Dis 29(6):850–65. 10.1093/ibd/izac266 36715181 10.1093/ibd/izac266

[74] FAURE-ANDRé G, Vargas P, Yuseff MI et al (2008) Regulation of dendritic cell migration by CD74, the MHC class II-associated invariant chain. Science (New York, NY) 322(5908):1705–10. 10.1126/science.1159894 10.1126/science.1159894 19074353

[75] Chai EZ, Siveen KS, Shanmugam MK et al (2015) Analysis of the intricate relationship between chronic inflammation and cancer. Biochem J 468(1):1–15. 10.1042/bj20141337 25940732 10.1042/BJ20141337

[76] Mantovani A, Garlanda C, Allavena P (2010) Molecular pathways and targets in cancer-related inflammation. Ann Med 42(3):161–70. 10.3109/07853890903405753 20384432 10.3109/07853890903405753

[77] Hanahan D, Weinberg RA (2011) Hallmarks of cancer: the next generation. Cell 144(5):646–74. 10.1016/j.cell.2011.02.013 21376230 10.1016/j.cell.2011.02.013

[78] Morton DL, Thompson JF, Cochran AJ et al (2014) Final trial report of sentinel-node biopsy versus nodal observation in melanoma. N Engl J Med 370(7):599–609. 10.1056/NEJMoa1310460 24521106 10.1056/NEJMoa1310460 PMC4058881

[79] Zahoor S, Haji A, Battoo A et al (2017) Sentinel lymph node biopsy in breast cancer: a clinical review and update. J Breast Cancer 20(3):217–27. 10.4048/jbc.2017.20.3.217 28970846 10.4048/jbc.2017.20.3.217 PMC5620435

[80] Stacker SA, Williams SP, Karnezis T et al (2014) Lymphangiogenesis and lymphatic vessel remodelling in cancer. Nat Rev Cancer 14(3):159–72. 10.1038/nrc3677 24561443 10.1038/nrc3677

[81] Dieterich LC, Tacconi C, Ducoli L et al (2022) Lymphatic vessels in cancer. Physiol Rev 102(4):1837–79. 10.1152/physrev.00039.2021 35771983 10.1152/physrev.00039.2021

[82] Lund AW, Wagner M, Fankhauser M et al (2016) Lymphatic vessels regulate immune microenvironments in human and murine melanoma. J Clin Invest 126(9):3389–402. 10.1172/jci79434 27525437 10.1172/JCI79434 PMC5004967

[83] Song E, Mao T, Dong H et al (2020) VEGF-C-driven lymphatic drainage enables immunosurveillance of brain tumours. Nature 577(7792):689–94. 10.1038/s41586-019-1912-x 31942068 10.1038/s41586-019-1912-x PMC7100608

[84] Lund AW, Duraes FV, Hirosue S et al (2012) VEGF-C promotes immune tolerance in B16 melanomas and cross-presentation of tumor antigen by lymph node lymphatics. Cell Rep 1(3):191–9. 10.1016/j.celrep.2012.01.005 22832193 10.1016/j.celrep.2012.01.005

[85] Roberts EW, Broz ML, Binnewies M et al (2016) Critical role for CD103(+)/CD141(+) dendritic cells bearing CCR7 for tumor antigen trafficking and priming of T Cell immunity in melanoma. Cancer Cell 30(2):324–36. 10.1016/j.ccell.2016.06.003 27424807 10.1016/j.ccell.2016.06.003 PMC5374862

[86] O’melia MJ, Rohner NA, Manspeaker MP et al (2020) Quality of CD8(+) T cell immunity evoked in lymph nodes is compartmentalized by route of antigen transport and functional in tumor context. Science advances. 10.1126/sciadv.abd7134 33310857 10.1126/sciadv.abd7134 PMC7732197

[87] Broggi MAS, Maillat L, Clement CC et al (2019) Tumor-associated factors are enriched in lymphatic exudate compared to plasma in metastatic melanoma patients. J Exp Med 216(5):1091–107. 10.1084/jem.20181618 30975896 10.1084/jem.20181618 PMC6504224

[88] Hirosue S, Vokali E, Raghavan VR et al (2014) Steady-state antigen scavenging, cross-presentation, and CD8 + T cell priming: a new role for lymphatic endothelial cells. Journal of immunology (Baltimore, Md : 1950) 192(11):5002–11. 10.4049/jimmunol.1302492 24795456 10.4049/jimmunol.1302492 PMC4025611

[89] Karakousi T, Mudianto T, Lund AW (2024) Lymphatic vessels in the age of cancer immunotherapy. Nat Rev Cancer 24(6):363–81. 10.1038/s41568-024-00681-y 38605228 10.1038/s41568-024-00681-y PMC12312704

[90] Christiansen AJ, Dieterich LC, Ohs I et al (2016) Lymphatic endothelial cells attenuate inflammation via suppression of dendritic cell maturation. Oncotarget 7(26):39421–35. 10.18632/oncotarget.9820 27270646 10.18632/oncotarget.9820 PMC5129942

[91] You S, Li S, Zeng L et al (2024) Lymphatic-localized Treg-mregDC crosstalk limits antigen trafficking and restrains anti-tumor immunity. Cancer Cell 42(8):1415–33.e12. 10.1016/j.ccell.2024.06.014 39029466 10.1016/j.ccell.2024.06.014

[92] Badillo O, Helfridsson L, Niemi J et al (2024) Exploring dendritic cell subtypes in cancer immunotherapy: unraveling the role of mature regulatory dendritic cells. Upsala journal of medical sciences. 10.48101/ujms.v129.10627 38716077 10.48101/ujms.v129.10627 PMC11075441

[93] KVEDARAite E, Ginhoux F (2022) Human dendritic cells in cancer. Sci Immunol 7(70):eabm9409. 10.1126/sciimmunol.abm9409 35363544 10.1126/sciimmunol.abm9409

[94] Neeland MR, Meeusen EN, DE Veer MJ (2014) Afferent lymphatic cannulation as a model system to study innate immune responses to infection and vaccination. Vet Immunol Immunopathol 158(1–2):86–97. 10.1016/j.vetimm.2013.01.004 23369582 10.1016/j.vetimm.2013.01.004

[95] Maisel K, Sasso MS, Potin L et al (2017) Exploiting lymphatic vessels for immunomodulation: rationale, opportunities, and challenges. Adv Drug Deliv Rev 114:43–59. 10.1016/j.addr.2017.07.005 28694027 10.1016/j.addr.2017.07.005 PMC6026542

[96] Tomura M, Honda T, Tanizaki H et al (2010) Activated regulatory T cells are the major T cell type emigrating from the skin during a cutaneous immune response in mice. J Clin Invest 120(3):883–93. 10.1172/jci40926 20179354 10.1172/JCI40926 PMC2827959

[97] Yawalkar N, Hunger RE, Pichler WJ et al (2000) Human afferent lymph from normal skin contains an increased number of mainly memory / effector CD4(+) T cells expressing activation, adhesion and co-stimulatory molecules. Eur J Immunol 30(2):491–7. 10.1002/1521-4141(200002)30:2<;491::Aid-immu491>;3.0.Co;2-h 10.1002/1521-4141(200002)30:2<491::AID-IMMU491>3.0.CO;2-H 10671204

[98] Bromley SK, Yan S, Tomura M et al (2013) Recirculating memory T cells are a unique subset of CD4 + T cells with a distinct phenotype and migratory pattern. Journal of immunology (Baltimore, Md : 1950) 190(3):970–6. 10.4049/jimmunol.1202805 23255361 10.4049/jimmunol.1202805 PMC3618989

[99] Tomura M, Yoshida N, Tanaka J et al (2008) Monitoring cellular movement in vivo with photoconvertible fluorescence protein “Kaede” transgenic mice. Proc Natl Acad Sci U S A 105(31):10871–6. 10.1073/pnas.0802278105 18663225 10.1073/pnas.0802278105 PMC2504797

[100] Nakanishi Y, Ikebuchi R, Chtanova T et al (2018) Regulatory T cells with superior immunosuppressive capacity emigrate from the inflamed colon to draining lymph nodes. Mucosal Immunol 11(2):437–48. 10.1038/mi.2017.64 28766553 10.1038/mi.2017.64

[101] LAL LEDGERWOODLG, ZHANG G (2008) The sphingosine 1-phosphate receptor 1 causes tissue retention by inhibiting the entry of peripheral tissue T lymphocytes into afferent lymphatics [J]. Nat Immunol 9(1):42–53. 10.1038/ni1534 18037890 10.1038/ni1534

[102] Skon CN, Lee JY, Anderson KG et al (2013) Transcriptional downregulation of S1pr1 is required for the establishment of resident memory CD8 + T cells. Nat Immunol 14(12):1285–93. 10.1038/ni.2745 24162775 10.1038/ni.2745 PMC3844557

[103] Salmi M, Koskinen K, Henttinen T et al (2004) CLEVER-1 mediates lymphocyte transmigration through vascular and lymphatic endothelium. Blood 104(13):3849–57. 10.1182/blood-2004-01-0222 15297319 10.1182/blood-2004-01-0222

[104] Tadayon S, Dunkel J, Takeda A et al (2021) Lymphatic endothelial cell activation and dendritic cell transmigration is modified by genetic deletion of Clever-1. Frontiers in immunology 12:602122. 10.3389/fimmu.2021.602122 33746947 10.3389/fimmu.2021.602122 PMC7970002

[105] Dubrot J, Duraes FV, Potin L et al (2014) Lymph node stromal cells acquire peptide-MHCII complexes from dendritic cells and induce antigen-specific CD4⁺ T cell tolerance. J Exp Med 211(6):1153–66. 10.1084/jem.20132000 24842370 10.1084/jem.20132000 PMC4042642

[106] Berendam SJ, Koeppel AF, Godfrey NR et al (2019) Comparative transcriptomic analysis identifies a range of immunologically related functional elaborations of lymph node associated lymphatic and blood endothelial cells. Frontiers in immunology 10:816. 10.3389/fimmu.2019.00816 31057546 10.3389/fimmu.2019.00816 PMC6478037

[107] Tewalt EF, Cohen JN, Rouhani SJ et al (2012) Lymphatic endothelial cells induce tolerance via PD-L1 and lack of costimulation leading to high-level PD-1 expression on CD8 T cells. Blood 120(24):4772–82. 10.1182/blood-2012-04-427013 22993390 10.1182/blood-2012-04-427013 PMC3520619

[108] Rouhani SJ, Eccles JD, Riccardi P et al (2015) Roles of lymphatic endothelial cells expressing peripheral tissue antigens in CD4 T-cell tolerance induction. Nature communications 6:6771. 10.1038/ncomms7771 25857745 10.1038/ncomms7771 PMC4403767

[109] Humbert M, Hugues S, Dubrot J (2016) Shaping of peripheral T cell responses by lymphatic endothelial cells. Frontiers in immunology 7:684. 10.3389/fimmu.2016.00684 28127298 10.3389/fimmu.2016.00684 PMC5226940

[110] Tamburini BA, Burchill MA, Kedl RM (2014) Antigen capture and archiving by lymphatic endothelial cells following vaccination or viral infection. Nature communications 5:3989. 10.1038/ncomms4989 24905362 10.1038/ncomms4989 PMC4073648

[111] Dieterich LC, Ikenberg K, Cetintas T et al (2017) Tumor-associated lymphatic vessels upregulate PDL1 to inhibit T-cell activation. Frontiers in immunology 8:66. 10.3389/fimmu.2017.00066 28217128 10.3389/fimmu.2017.00066 PMC5289955

[112] Brown MN, Fintushel SR, Lee MH et al (2010) Chemoattractant receptors and lymphocyte egress from extralymphoid tissue: changing requirements during the course of inflammation. Journal of immunology (Baltimore, Md : 1950) 185(8):4873–82. 10.4049/jimmunol.1000676 20833836 10.4049/jimmunol.1000676 PMC3327166

[113] Gao D, Zou B, Zhu K et al (2024) Enhancing Th17 cells drainage through meningeal lymphatic vessels alleviate neuroinflammation after subarachnoid hemorrhage. J Neuroinflammation 21(1):269. 10.1186/s12974-024-03252-y 39428510 10.1186/s12974-024-03252-y PMC11492769

[114] Lerner TR, DE Souza Carvalho-Wodarz C, Repnik U et al (2016) Lymphatic endothelial cells are a replicative niche for *Mycobacterium tuberculosis*. J Clin Invest 126(3):1093–108. 10.1172/jci83379 26901813 10.1172/JCI83379 PMC4767353

[115] Schilthuis M, Verkaik S, Walhof M et al (2018) Lymphatic endothelial cells promote productive and latent HIV infection in resting CD4 + T cells. Virol J 15(1):152. 10.1186/s12985-018-1068-6 30285810 10.1186/s12985-018-1068-6 PMC6169068

[116] Louveau A, DA Mesquita S, Kipnis J (2016) Lymphatics in neurological disorders: a neuro-lympho-vascular component of Multiple Sclerosis and Alzheimer’s Disease? Neuron 91(5):957–73. 10.1016/j.neuron.2016.08.027 27608759 10.1016/j.neuron.2016.08.027 PMC5019121

[117] Kallal N, Hugues S, Garnier L (2024) Regulation of autoimmune-mediated neuroinflammation by endothelial cells. Eur J Immunol 54(4):e2350482. 10.1002/eji.202350482 38335316 10.1002/eji.202350482

[118] Garnier L, Gkountidi AO, Hugues S (2019) Tumor-associated lymphatic vessel features and immunomodulatory functions. Front Immunol 10:720. 10.3389/fimmu.2019.00720 31024552 10.3389/fimmu.2019.00720 PMC6465591

[119] Chen DS, Mellman I (2017) Elements of cancer immunity and the cancer-immune set point. Nature 541(7637):321–30. 10.1038/nature21349 28102259 10.1038/nature21349

[120] Liu YT, Sun ZJ (2021) Turning cold tumors into hot tumors by improving T-cell infiltration. Theranostics 11(11):5365–86. 10.7150/thno.58390 33859752 10.7150/thno.58390 PMC8039952

[121] STEELE M M, CHURCHILL M J, BREAZEALE A P et al (2019) Quantifying Leukocyte Egress via Lymphatic Vessels from Murine Skin and Tumors [J]. J visualized experiments: JoVE 143. 10.3791/58704 10.3791/58704 30663703

[122] Chen IX, Chauhan VP, Posada J et al (2019) Blocking CXCR4 alleviates desmoplasia, increases T-lymphocyte infiltration, and improves immunotherapy in metastatic breast cancer. Proc Natl Acad Sci U S A 116(10):4558–66. 10.1073/pnas.1815515116 30700545 10.1073/pnas.1815515116 PMC6410779

[123] Chen Y, Ramjiawan RR, Reiberger T et al (2015) CXCR4 inhibition in tumor microenvironment facilitates anti-programmed death receptor-1 immunotherapy in sorafenib-treated hepatocellular carcinoma in mice. Hepatology 61(5):1591–602. 10.1002/hep.27665 25529917 10.1002/hep.27665 PMC4406806

[124] Fridman WH, PAGèS F, SAUTèS-FRIDMAN C et al (2012) The immune contexture in human tumours: impact on clinical outcome. Nat Rev Cancer 12(4):298–306. 10.1038/nrc3245 22419253 10.1038/nrc3245

[125] Saleh R, Elkord E (2020) FoxP3(+) T regulatory cells in cancer: prognostic biomarkers and therapeutic targets. Cancer Lett 490:174–85. 10.1016/j.canlet.2020.07.022 32721551 10.1016/j.canlet.2020.07.022

[126] HUANG C H, KU W T, MAHALINGAM J et al (2025) Tumor-migrating peripheral Foxp3-high regulatory T cells drive poor prognosis in hepatocellular carcinoma [J]. Hepatology (Baltimore MD). 10.1097/hep.0000000000001428 40590861 10.1097/HEP.0000000000001428

[127] Watson MJ, Vignali PDA, Mullett SJ et al (2021) Metabolic support of tumour-infiltrating regulatory T cells by lactic acid. Nature 591(7851):645–51. 10.1038/s41586-020-03045-2 33589820 10.1038/s41586-020-03045-2 PMC7990682

[128] Zhu L, Bai Y, Li A et al (2024) IFN-γ-responsiveness of lymphatic endothelial cells inhibits melanoma lymphatic dissemination via AMPK-mediated metabolic control. Biochim Biophys Acta Mol Basis Dis 1870(7):167314. 10.1016/j.bbadis.2024.167314 38936516 10.1016/j.bbadis.2024.167314

[129] Sokolowski J, Jakobsen E, Johannessen JV (1978) Cells in peripheral leg lymph of normal men. Lymphology 11(4):202–7 739794

[130] Platt AM, Randolph GJ (2013) Cellular Composition of Lymph. In: Santambrogio L (ed) Immunology of the Lymphatic System. Springer New York, New York, NY, pp 53–64

[131] Ebisuno Y, Tanaka T, Kanemitsu N et al (2003) Cutting edge: the b cell chemokine CXC chemokine ligand 13/b lymphocyte chemoattractant is expressed in the high endothelial venules of lymph nodes and Peyer’s patches and affects b cell trafficking across high endothelial venules. Journal of immunology (Baltimore, Md : 1950) 171(4):1642–6. 10.4049/jimmunol.171.4.1642 12902460 10.4049/jimmunol.171.4.1642

[132] YANG J, ZHANG S, ZHANG L et al (2019) Lymphatic endothelial cells regulate B-cell homing to lymph nodes via a NIK-dependent mechanism [J]. Cell Mol Immunol 16(2):165–177. 10.1038/cmi.2017.167 29503445 10.1038/cmi.2017.167 PMC6355805

[133] Shrestha B, Hashiguchi T, Ito T et al (2010) B cell-derived vascular endothelial growth factor A promotes lymphangiogenesis and high endothelial venule expansion in lymph nodes. Journal of immunology (Baltimore, Md : 1950) 184(9):4819–26. 10.4049/jimmunol.0903063 20308631 10.4049/jimmunol.0903063

[134] Dubey LK, Karempudi P, Luther SA et al (2017) Interactions between fibroblastic reticular cells and b cells promote mesenteric lymph node lymphangiogenesis. Nat Commun 8(1):367. 10.1038/s41467-017-00504-9 28848229 10.1038/s41467-017-00504-9 PMC5573728

[135] Hampton HR, Bailey J, Tomura M et al (2015) Microbe-dependent lymphatic migration of neutrophils modulates lymphocyte proliferation in lymph nodes. Nat Commun 6:7139. 10.1038/ncomms8139 25972253 10.1038/ncomms8139 PMC4479041

[136] Stephens M, Liao S (2018) Neutrophil-lymphatic interactions during acute and chronic disease. Cell Tissue Res 371(3):599–606. 10.1007/s00441-017-2779-5 29423716 10.1007/s00441-017-2779-5

[137] Gorlino CV, Ranocchia RP, Harman MF et al (2014) Neutrophils exhibit differential requirements for homing molecules in their lymphatic and blood trafficking into draining lymph nodes. Journal of immunology (Baltimore, Md : 1950) 193(4):1966–74. 10.4049/jimmunol.1301791 25015824 10.4049/jimmunol.1301791

[138] Tan KW, Chong SZ, Wong FH et al (2013) Neutrophils contribute to inflammatory lymphangiogenesis by increasing VEGF-A bioavailability and secreting VEGF-D. Blood 122(22):3666–77. 10.1182/blood-2012-11-466532 24113869 10.1182/blood-2012-11-466532

[139] Ning Y, Chen Y, Tian T et al (2024) S100A7 orchestrates neutrophil chemotaxis and drives neutrophil extracellular traps (NETs) formation to facilitate lymph node metastasis in cervical cancer patients. Cancer Lett 605:217288. 10.1016/j.canlet.2024.217288 39384116 10.1016/j.canlet.2024.217288

[140] Bogoslowski A, Butcher EC, Kubes P (2018) Neutrophils recruited through high endothelial venules of the lymph nodes via PNAd intercept disseminating *Staphylococcus aureus*. Proc Natl Acad Sci U S A 115(10):2449–54. 10.1073/pnas.1715756115 29378967 10.1073/pnas.1715756115 PMC5877924

[141] BöHMER R, Neuhaus B, BüHREN S et al (2010) Regulation of developmental lymphangiogenesis by Syk(+) leukocytes. Developmental cell 18(3):437–49. 10.1016/j.devcel.2010.01.009 20230750 10.1016/j.devcel.2010.01.009

[142] LEIRIãO P, Del Fresno C, ARDAVíN C (2012) Monocytes as effector cells: activated Ly-6C(high) mouse monocytes migrate to the lymph nodes through the lymph and cross-present antigens to CD8 + T cells. European journal of immunology 42(8):2042–51. 10.1002/eji.201142166 22585535 10.1002/eji.201142166

[143] Haemmerle M, Keller T, Egger G et al (2013) Enhanced lymph vessel density, remodeling, and inflammation are reflected by gene expression signatures in dermal lymphatic endothelial cells in type 2 diabetes. Diabetes 62(7):2509–29. 10.2337/db12-0844 23423575 10.2337/db12-0844 PMC3712036

[144] Kang S, Lee SP, Kim KE et al (2009) Toll-like receptor 4 in lymphatic endothelial cells contributes to LPS-induced lymphangiogenesis by chemotactic recruitment of macrophages. Blood 113(11):2605–13. 10.1182/blood-2008-07-166934 19098273 10.1182/blood-2008-07-166934

[145] Buttler K, Kreysing A, VON Kaisenberg CS et al (2006) Mesenchymal cells with leukocyte and lymphendothelial characteristics in murine embryos. Dev Dyn 235(6):1554–62. 10.1002/dvdy.20737 16502417 10.1002/dvdy.20737

[146] Hall KL, Volk-Draper LD, Flister MJ et al (2012) New model of macrophage acquisition of the lymphatic endothelial phenotype. PLoS One 7(3):e31794. 10.1371/journal.pone.0031794 22396739 10.1371/journal.pone.0031794 PMC3292559

[147] Srinivasan RS, Dillard ME, Lagutin OV et al (2007) Lineage tracing demonstrates the venous origin of the mammalian lymphatic vasculature. Genes Dev 21(19):2422–32. 10.1101/gad.1588407 17908929 10.1101/gad.1588407 PMC1993873

[148] Guo H, Liu Z, Lv R et al (2025) Aldosterone induces renal lymphangiogenesis through macrophage-lymphatic endothelial cell transformation and inhibition by esaxerenone. Inflamm Res 74(1):85. 10.1007/s00011-025-02044-1 40413281 10.1007/s00011-025-02044-1

[149] Schledzewski K, Falkowski M, Moldenhauer G et al (2006) Lymphatic endothelium-specific hyaluronan receptor LYVE-1 is expressed by stabilin-1+, F4/80+, CD11b+ macrophages in malignant tumours and wound healing tissue in vivo and in bone marrow cultures in vitro: implications for the assessment of lymphangiogenesis. J Pathol 209(1):67–77. 10.1002/path.1942 16482496 10.1002/path.1942

[150] Ran S, Volk-Draper L (2020) Lymphatic endothelial cell progenitors in the tumor microenvironment. Adv Exp Med Biol 1234:87–105. 10.1007/978-3-030-37184-5_7 32040857 10.1007/978-3-030-37184-5_7 PMC7263265

[151] Bessell E, Finlay RE, James LK et al (2024) Stromal cell and b cell dialogue potentiates IL-33-enriched lymphoid niches to support eosinophil recruitment and function during type 2 immunity. Cell Rep 43(8):114620. 10.1016/j.celrep.2024.114620 39141517 10.1016/j.celrep.2024.114620

[152] Ruddle NH (2014) Lymphatic vessels and tertiary lymphoid organs. J Clin Invest 124(3):953–9. 10.1172/jci71611 24590281 10.1172/JCI71611 PMC3934190

[153] Ruddle NH (2016) High endothelial venules and lymphatic vessels in tertiary lymphoid organs: characteristics, functions, and regulation. Front Immunol 7:491. 10.3389/fimmu.2016.00491 27881983 10.3389/fimmu.2016.00491 PMC5101196

[154] Randolph GJ, Bala S, Rahier JF et al (2016) Lymphoid aggregates remodel lymphatic collecting vessels that serve mesenteric lymph nodes in Crohn disease. Am J Pathol 186(12):3066–73. 10.1016/j.ajpath.2016.07.026 27746181 10.1016/j.ajpath.2016.07.026 PMC5225286

[155] Erlich EC, Alayo QA, Kim A et al (2025) Distinct roles for B cell-derived LTα3 and LTα1β2 in TNF-mediated ileitis. Nat Immunol 26(10):1781–93. 10.1038/s41590-025-02263-y 40921841 10.1038/s41590-025-02263-y PMC12479355

[156] Lok LSC, Dennison TW, Mahbubani KM et al (2019) Phenotypically distinct neutrophils patrol uninfected human and mouse lymph nodes. Proc Natl Acad Sci U S A 116(38):19083–9. 10.1073/pnas.1905054116 31484769 10.1073/pnas.1905054116 PMC6754587

[157] Bogoslowski A, Wijeyesinghe S, Lee WY et al (2020) Neutrophils recirculate through lymph nodes to survey tissues for pathogens. Journal of immunology (Baltimore, Md : 1950) 204(9):2552–61. 10.4049/jimmunol.2000022 32205425 10.4049/jimmunol.2000022

[158] Herro R, Grimes HL (2024) The diverse roles of neutrophils from protection to pathogenesis. Nat Immunol 25(12):2209–19. 10.1038/s41590-024-02006-5 39567761 10.1038/s41590-024-02006-5

[159] Kolaczkowska E, Kubes P (2013) Neutrophil recruitment and function in health and inflammation. Nat Rev Immunol 13(3):159–75. 10.1038/nri3399 23435331 10.1038/nri3399

[160] Wang J, Hossain M, Thanabalasuriar A et al (2017) Visualizing the function and fate of neutrophils in sterile injury and repair. Science (New York, NY) 358(6359):111–6. 10.1126/science.aam9690 10.1126/science.aam9690 28983053

[161] Rigby DA, Ferguson DJ, Johnson LA et al (2015) Neutrophils rapidly transit inflamed lymphatic vessel endothelium via integrin-dependent proteolysis and lipoxin-induced junctional retraction. J Leukoc Biol 98(6):897–912. 10.1189/jlb.1HI0415-149R 26216937 10.1189/jlb.1HI0415-149R

[162] Arokiasamy S, Zakian C, Dilliway J et al (2017) Endogenous TNFα orchestrates the trafficking of neutrophils into and within lymphatic vessels during acute inflammation. Sci Rep 7:44189. 10.1038/srep44189 28287124 10.1038/srep44189 PMC5347029

[163] Poto R, Loffredo S, Palestra F et al (2022) Angiogenesis, lymphangiogenesis, and inflammation in chronic obstructive pulmonary disease (COPD): few certainties and many outstanding questions. Cells. 10.3390/cells11101720 35626756 10.3390/cells11101720 PMC9139415

[164] Lu Q, Yin H, Deng Y et al (2022) circDHTKD1 promotes lymphatic metastasis of bladder cancer by upregulating CXCL5. Cell Death Discov 8(1):243. 10.1038/s41420-022-01037-x 35504887 10.1038/s41420-022-01037-x PMC9065127

[165] Lavoie SS, Dumas E, Vulesevic B et al (2018) Synthesis of human neutrophil extracellular traps contributes to angiopoietin-mediated in vitro proinflammatory and proangiogenic activities. J Immunol 200(11):3801–13. 10.4049/jimmunol.1701203 29686052 10.4049/jimmunol.1701203

[166] Yang S, Sun B, Li J et al (2023) Neutrophil extracellular traps promote angiogenesis in gastric cancer. Cell Commun Signal 21(1):176. 10.1186/s12964-023-01196-z 37480055 10.1186/s12964-023-01196-z PMC10362668

[167] GLINTON K E, MA W (2022) Macrophage-produced VEGFC is induced by efferocytosis to ameliorate cardiac injury and inflammation [J]. J Clin Investig 132(9). 10.1172/jci140685 10.1172/JCI140685 PMC9057589 35271504

[168] Becker F, Kurmaeva E, Gavins FN et al (2016) A critical role for monocytes/macrophages during intestinal inflammation-associated lymphangiogenesis. Inflamm Bowel Dis 22(6):1326–45. 10.1097/mib.0000000000000731 26950310 10.1097/MIB.0000000000000731 PMC4912566

[169] GULA G, NIDERLA-BIELIŃSKA J RUMIŃSKIS et al (2021) Potential functions of embryonic cardiac macrophages in angiogenesis, lymphangiogenesis and extracellular matrix remodeling [J]. Histochem Cell Biol 155(1):117–132. 10.1007/s00418-020-01934-1 33130914 10.1007/s00418-020-01934-1 PMC7847984

[170] Kubota Y, Takubo K, Shimizu T et al (2009) M-CSF inhibition selectively targets pathological angiogenesis and lymphangiogenesis. Journal of Experimental Medicine 206(5):1089–102. 10.1084/jem.20081605 19398755 10.1084/jem.20081605 PMC2715025

[171] Kataru RP, Jung K, Jang C et al (2009) Critical role of CD11b+ macrophages and VEGF in inflammatory lymphangiogenesis, antigen clearance, and inflammation resolution. Blood 113(22):5650–9. 10.1182/blood-2008-09-176776 19346498 10.1182/blood-2008-09-176776

[172] Lee KM, Danuser R, Stein JV et al (2014) The chemokine receptors ACKR2 and CCR2 reciprocally regulate lymphatic vessel density. The EMBO journal 33(21):2564–80. 10.15252/embj.201488887 25271254 10.15252/embj.201488887 PMC4283412

[173] Nakamoto S, Ito Y, Nishizawa N et al (2020) Lymphangiogenesis and accumulation of reparative macrophages contribute to liver repair after hepatic ischemia-reperfusion injury. Angiogenesis 23(3):395–410. 10.1007/s10456-020-09718-w 32162023 10.1007/s10456-020-09718-w

[174] GHANTA S, CUZZONE D A, TORRISI JS et al (2015) Regulation of inflammation and fibrosis by macrophages in lymphedema [J]. Am J Physiol Heart Circ Physiol 308(9):H1065–H1077. 10.1152/ajpheart.00598.2014 25724493 PMC4551121

[175] Conrad C, Niess H, Huss R et al (2009) Multipotent mesenchymal stem cells acquire a lymphendothelial phenotype and enhance lymphatic regeneration in vivo. Circulation 119(2):281–9. 10.1161/circulationaha.108.793208 19118255 10.1161/CIRCULATIONAHA.108.793208

[176] Du Y, Cao M, Liu Y et al (2022) Tumor microenvironment remodeling modulates macrophage phenotype in breast cancer lymphangiogenesis. FASEB J 36(4):e22248. 10.1096/fj.202101230R 35239213 10.1096/fj.202101230R

[177] Petkova M, Kraft M, Stritt S et al (2023) Immune-interacting lymphatic endothelial subtype at capillary terminals drives lymphatic malformation. J Exp Med. 10.1084/jem.20220741 36688917 10.1084/jem.20220741 PMC9884640

[178] Doni A, Sironi M, Del Prete A et al (2024) PTX3 is expressed in terminal lymphatics and shapes their organization and function. Front Immunol 15:1426869. 10.3389/fimmu.2024.1426869 39640269 10.3389/fimmu.2024.1426869 PMC11617523

[179] Fernandes LM, Griswold-Wheeler D, Tresemer JD et al (2025) A single-cell atlas of normal and KRASG12D-malformed lymphatic vessels. JCI Insight. 10.1172/jci.insight.185181 39874106 10.1172/jci.insight.185181 PMC11949019

